# Digitaldlsorter: Deep-Learning on scRNA-Seq to Deconvolute Gene Expression Data

**DOI:** 10.3389/fgene.2019.00978

**Published:** 2019-10-25

**Authors:** Carlos Torroja, Fatima Sanchez-Cabo

**Affiliations:** Bioinformatics Unit, Fundación Centro Nacional de Investigaciones Cardiovasculares (CNIC), Madrid, Spain

**Keywords:** Machine learning, Deconvolution algorithm, Cancer, immunology, single-cell

## Abstract

The development of single cell transcriptome sequencing has allowed researchers the possibility to dig inside the role of the individual cell types in a plethora of disease scenarios. It also expands to the whole transcriptome what before was only possible for a few tenths of antibodies in cell population analysis. More importantly, it allows resolving the permanent question of whether the changes observed in a particular bulk experiment are a consequence of changes in cell type proportions or an aberrant behavior of a particular cell type. However, single cell experiments are still complex to perform and expensive to sequence making bulk RNA-Seq experiments yet more common. scRNA-Seq data is proving highly relevant information for the characterization of the immune cell repertoire in different diseases ranging from cancer to atherosclerosis. In particular, as scRNA-Seq becomes more widely used, new types of immune cell populations emerge and their role in the genesis and evolution of the disease opens new avenues for personalized immune therapies. Immunotherapy have already proven successful in a variety of tumors such as breast, colon and melanoma and its value in other types of disease is being currently explored. From a statistical perspective, single-cell data are particularly interesting due to its high dimensionality, overcoming the limitations of the “skinny matrix” that traditional bulk RNA-Seq experiments yield. With the technological advances that enable sequencing hundreds of thousands of cells, scRNA-Seq data have become especially suitable for the application of Machine Learning algorithms such as Deep Learning (DL). We present here a DL based method to enumerate and quantify the immune infiltration in colorectal and breast cancer bulk RNA-Seq samples starting from scRNA-Seq. Our method makes use of a Deep Neural Network (DNN) model that allows quantification not only of lymphocytes as a general population but also of specific CD8+, CD4Tmem, CD4Th and CD4Tregs subpopulations, as well as B-cells and Stromal content. Moreover, the signatures are built from scRNA-Seq data from the tumor, preserving the specific characteristics of the tumor microenvironment as opposite to other approaches in which cells were isolated from blood. Our method was applied to synthetic bulk RNA-Seq and to samples from the TCGA project yielding very accurate results in terms of quantification and survival prediction.

## Introduction

During the last two decades, since the discovery that immune cells play a key role in tumor progression (Staveley-O’Carroll et al., 1998), much effort has been done into the identification and quantification of the different immune cell types infiltrating the tumor (TILs) and its relationship with the prognosis of the patients. First findings in colorectal cancer (Galon et al., 2006) demonstrated that the presence of TILs correlated positively with the prognosis of the patient. The immunoscore proposed in this work has been validated in several cohorts all around the world and is undergoing several clinical trials for its transfer into the clinical practice (Ogino and Giannakis, 2018). More complex relationships have been also found for different types of TILs, with some pro-tumoral types like T-Regs, M2 Macrophages, pDCs or some types of B-cells and others anti-tumoral like CD8+ T-cells, some T-Helpers cells, NKs and Ig secreting B-cells (Bingle et al., 2002; Jahrsdörfer et al., 2010; Sarvaria et al., 2017). Moreover, the action of the tumor context into the plasticity of several types of lymphocytes makes these relationships more complex and difficult to extrapolate from one tumor to another (Colbeck et al., 2017).

Antibodies designed to interfere with immune response modulators like the immune-suppressive receptors Cytotoxic T-Lymphocyte Antigen 4 (CTLA-4) and Programmed Cell Death 1 (PD1) (Seidel et al., 2018) or costimulatory receptors like Tumor Necrosis Factor Receptor Superfamily Member 18 (TNFRSF18 or GITR) or Tumor Necrosis Factor Receptor Superfamily Member 4 (TNFRSF4 or OX40) are going through clinical trials with striking results although in a few proportion of patients. Many experiments suggest that this lack of generalization is dependent on the presence, in the tumor microenvironment, of activating Fc receptors expressed on myeloid cells that will induce CD4 T-Reg cells depletion through antibody-dependent cellular cytotoxicity (Furness et al., 2014). Several strategies are being develop also to interfere with the pro-tumoral functions of Macrophages and Monocytes mainly through combinations of agonist to CD40 to active anti-tumoral functions and anti-CSF-R1 (Colony Stimulating Factor 1 Receptor) to reduce their immune suppressive functions (Guerriero, 2018). Moreover, the action of the tumor context into the plasticity of several types of lymphocytes makes these relationships more complex and difficult to extrapolate from one tumor to another (Colbeck et al., 2017).

Traditional approaches for the characterization of immune cell populations at the protein level tried to combine immunohistochemistry (IHC), immune fluorescence (IF), and flow cytometry followed by manual and/or complex image analysis algorithms. These approaches are to date the most precise ones allowing not only the definition and quantification of specific TIL subpopulations but also their localization within the tumor. These three parameter have proven essential to predict overall and disease-free survival rates (Pagès et al., 2018). As discussed before, patient response to treatments depends on the particular functional status and distribution of the different cell types in the tumor microenvironment. These protein based methods are not able to interrogate large amounts of profiles and therefore lack some precision to characterize the functional status of the cells. Besides, these procedures require a lot of manipulation, and the analysis is very time consuming making them not suitable for large-scale.

To overcome these limitations Next Generation Sequencing based transcriptomics (RNA-Seq) has emerged in the last years as a very promising methodology to explore. It is cheap, reproducible, robust, and scalable. The strength of the protocols for data generation and data analysis and the fact that it produces quantitative data versus other more qualitative technologies such as proteomics, makes it an ideal technology to be used in precision medicine (Collins and Varmus, 2015). The ability to capture the expression profile of many genes at the same time makes it very suitable to characterize many cell populations and their functional status at the same time.

Many computational or in-silico tools have been developed so far to get insight into the tumor cell composition by analyzing the transcriptional profile of a biopsy sample, such as CIBERSORT (Newman et al., 2015), EnrichR (Kuleshov et al., 2016), TIMER (Li et al., 2017a), EPIC (Racle et al., 2017), ESTIMATE (Yoshihara et al., 2013)or MCPCounter (Becht et al., 2016)among others (Finotello and Trajanoski, 2018). The goal is to identify how much the transcriptome of each particular cell type contributes to the overall bulk transcriptome of the sample. Some algorithms try to obtain TILs proportions by analyzing the enrichment of sets of predefined markers specific of each cell type (Kuleshov et al., 2016). Others, in turn, use different mathematical models to deconvolute the bulk gene expression into the expression from each cell population present in the sample (Newman et al., 2015; Li et al., 2017a). Both approaches rely on sets of markers or on whole transcriptome profiles obtained from purified or enriched samples from different sources. Although in principle they can be used in different contexts, their accuracy is limited by the fact that tumor, stromal and immune cells change significantly their profiles depending on the tissue and disease context (Cohen and Blasberg, 2017; Berglund et al., 2018; Shin et al., 2018).

Recent single cell RNA-Seq (scRNA-Seq) studies confirm the impact of the microenvironment in the transcriptome (Azizi et al., 2018; Bartoschek et al., 2018; Zemmour et al., 2018). Ideally, a deconvolution method should hence use gene signature profiles from cells obtained from the tissue or tumor to analyze, including not only the immune cells but also stromal and tumor cells themselves. This would allow a more precise quantification and definition of the physiological status of the cells (Mao et al., 2013). Following this idea, scRNA-Seq is being currently used massively to get a more precise understanding of the different cell populations present in different types of tumors, from breast (Chung et al., 2017), melanoma (Tirosh et al., 2016) and colorectal cancer (Li et al., 2017b). Unfortunately, this technology is yet relatively expensive and hence, available datasets have only sequenced a limited number of cells. However, the information they yield can be very useful to deconvolute gene expression from bulk samples (Baron et al., 2016), which is yet the preferred method from a practical point of view and for the investigation of hundreds of samples.

Up to date, the use of Machine Learning approaches beyond elastic net or random forest has not been possible for molecular data, due to the small number of samples available. scRNA-Seq changes this paradigm, increasing the sample size to hundreds of thousands of cells that can be understood as different samples from a statistical perspective. On top of that, techniques for oversampling (Chawla et al., 2002) are often used to amplify the signal in neural network approaches. For single cell data, several simulators are available (Risso et al., 2018) and we can use them to increase our sample size. This approach will work well if the original distribution of the data has been correctly inferred from the scRNA-Seq data available.

In this paper we present DigitalDLSorter, a DL based method to enumerate and quantify the cell type composition of a bulk RNA-Seq sample. Our method makes use of a Deep Neural Network (DNN) model to adjust any cell type composition defined from a scRNA-Seq data allowing the quantification not only of general cell types like lymphocytes but also of specific subpopulations and tissue dependent status. We have applied this method to evaluate the immune infiltration in colorectal and breast cancer bulk RNA-Seq samples starting from scRNA-Seq. The DigitalDLSorter model has been trained from scRNA-Seq data from the tumor itself, preserving the specific characteristics of the tumor microenvironment as opposite to other approaches in which cells were isolated from blood. Our method was applied to simulated bulk RNA-Seq and to samples generated by the TCGA Research Network (https://www.cancer.gov/tcga) yielding very accurate results in terms of quantification and survival prediction.

## Materials and Methods

### Datasets

To exemplify the digitalDLSorter pipeline and to explore its potential as a tool in tumor immunology we analyzed two single cell RNA-Seq experiments from the literature and stored in the Gene Expression Omnibus database (GEO: https://www.ncbi.nlm.nih.gov/geo/), one on breast cancer and one colorectal cancer experiment. The breast cancer (BC) experiment has 10 samples from different tumor etiology and stages (Chung et al., 2017) (**Table 1**, GSE75688) and the colorectal cancer (CRC) experiment has 11 samples (Li et al., 2017b) (**Table 2**, GSE81861).

**Table 1:** Samples from BC experiment. Subtype indicates the molecular characterization of the breast cancer based on the presence of HER2, PR and ER markers in the tumor cells (Dai et al., 2015).

Patient	Subtype	Tissue	Total Cells
**BC01**	luminalA	Breast	22
**BC02**	luminalA	Breast	53
**BC03**	luminalB	Breast	33
**BC04**	HER2	Breast	55
**BC05**	HER2	Breast	76
**BC06**	HER2	Breast	18
**BC07**	TNBC	Breast	50
**BC08**	TNBC	Breast	22
**BC09**	TNBC	Breast	55
**BC10**	TNBC	Breast	15
**BC11**	TNBC	Breast	11
**BC03**	luminalB	Lymphnode	53
**BC07**	TNBC	Lymphnode	52

HER2: ER-, PR-, HER2+ tumour cells.

LuminalA: ER+ and/or PR+, HER2- tumour cells.

LuminalB: ER+ and/or PR+, HER2+ tumour cells.

TNBC: ER- PR- and HER2-.

**Table 2:** Samples from CRC experiment.

Patient	Gender	TNM	Tumor_grade	Stage	Tumor Sample Cells	Normal Sample Cells	Total Cells
**CRC01**	Female	T4bN0M0	2	IIC	44	0	44
**CRC02**	Male	T3N1M0	1	IIIB	54	0	54
**CRC03**	Female	T4aN2M0	2	IIIB	17	7	24
**CRC04**	Female	T3N2M0	2	IIIB	18	18	36
**CRC05**	Female	T3N2M0	1	IIA	13	31	44
**CRC06**	Male	T3N2M0	2	IIIB	36	23	59
**CRC07**	Male	T3N0M0	2	IIA	34	0	34
**CRC08**	Female	T2N0M0	2	I	35	28	63
**CRC09**	Female	T3N2M0	2	IIIB	83	0	83
**CRC10**	Female	T3N0M0	2	IIA	29	62	91
**CRC11**	Male	T1N0M0	2	I	12	46	58

TNM, detailed tumor extension classification (T tumor, N lymph nodes, M metastasis); TUMOR GRADE, morphology based classification of tumor; STAGE, standard tumor extension classification; TUMOR SAMPLE CELLS, cells obtained from tumor biopsy; NORMAL SAMPLE CELLS, cells obtained from healthy paired tissue.

### scRNA-Seq Data Analysis

Both experiments were analyzed using Seurat (Butler et al., 2018) framework developed as an R package for clustering and representation purposes. We used the graph-based clustering approach implemented in Seurat to define the clusters and t-SNE dimensionality reduction (Maaten and Hinton, 2008) for visual representation of the cells.

To classify cells between tumor and non-tumor cells (stroma and immune cells) we used the RNA-Seq based CNV method described in (Chung et al., 2017). In the case of the presence of paired normal samples as in CRC experiment, tumor sample derived cells are considered as non-tumor when they cluster within clusters produced by normal tissue cells and using the cell type annotation itself.

To map the identified cells to its canonical cell type we used the xCell platform, both from web interface (http://xcell.ucsf.edu/) (Aran et al., 2017) for cluster average profiles, a more bulk like profile, and its R implementation specific for single cell classification, SingleR (Aran et al., 2017). We used the average profiles of the clusters together with the single cell profiles in both platforms and the manual inspection of typical cell type markers to label the different cell type groups identified in the experiment.

### DigitalDLSorter Pipeline Methods

The following sections describe the methodology used at the different steps of the DigitalDLSorter pipeline. The code to run the pipeline can be obtained at https://github.com/cartof/digitalDLSorter.

### Summary

**Figure 1:**
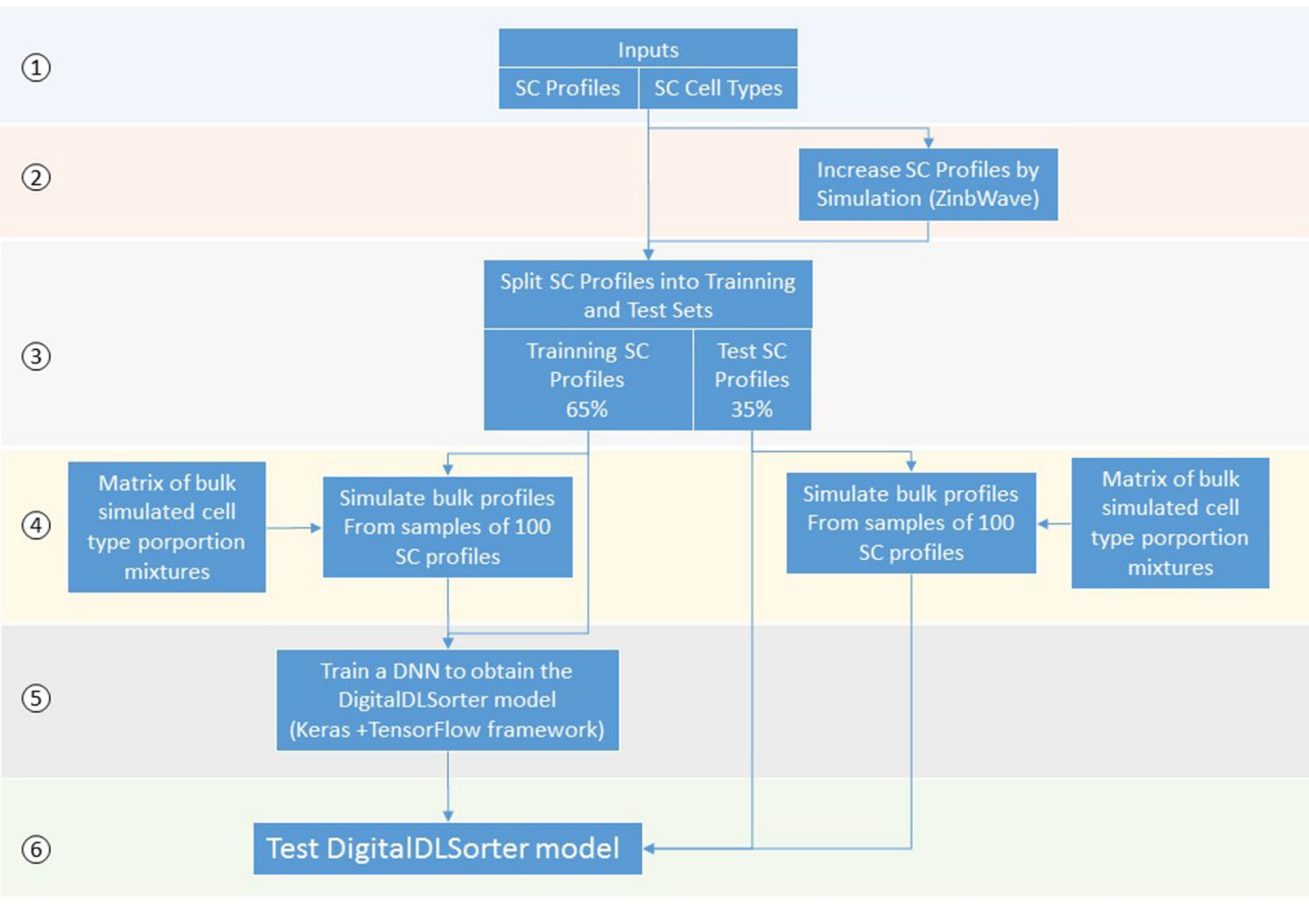
Scheme of the DigitalDLSorter Pipeline. 1) The pipeline takes a matrix of sc-RNASeq gene expression profiles (SC profiles) and a phenotype file indicating the cell type of each cell. 2) In those experiments were the number of SC profiles for each cell type is low, new SC profiles are simulated based on the real data using ZinbWave framework (Risso et al., 2018). 3) Real and simulated SC profiles are split into training (65%) and test (35%) sets. 4) For each training and test set, bulk samples are created by mixing 100 single cell profiles sampled according to the bulk cell type proportions simulated for each set. 5) A DNN is trained with the training set of bulk profiles together with the corresponding SC profiles. 6) The model obtained is applied to the test set, bulk and SC profiles.

DigitalDLSorter is a Deep Learning (DL) based method to quantify cell type proportions from bulk RNA-Seq samples as well as a classifier of single cell profiles (**Figure 1**). DigitalDLSorter pipeline starts from a matrix of single cell RNA-Seq profiles. If a given cell-type is not represented with at least 1000 gene expression profiles in the input data set, the input profiles are used to simulate new profiles of those under-represented. Then, the single cell profiles are split into a training (65%) and test set (35%) and used to generate synthetic bulk samples with known cell-type proportions for training and test respectively. The synthetic bulk and the single-cell profiles from the training dataset are used to train a deep neural network (DNN). The model obtained from the training is then applied to the test set to evaluate its performance on the prediction of different cell type proportions. The same model was applied to the TGCA data for real bulk performance evaluation.

### Oversampling of Low Frequency Cell Types

DL models need high dimensional data to achieve a good performance. Oversampling is a widely used approach to improve sample size in machine learning problems (Chawla et al., 2002). For underrepresented cell populations or for particularly small scRNA-Seq experiments we simulate single cell transcriptional profiles using available single cell simulation methods. In particular, we chose Zinb-wave (Risso et al., 2018) due to its ability to accommodate not only the variability within a particular cell type but also the variability within the whole experiment. First, we fed Zinb-wave with all the single cell profiles in the experiment, in order to estimate the parameters of a model. This includes the cell type and, as covariates, the sample of origin and the gender in the case of the CRC experiment (∼CellType+Sample+Gender), or the tissue of origin (breast or lymph node) in the case of the BC experiment (∼CellType+Sample+Tissue). Zinb-wave is also able to fit a model considering the gene length. However, as we are using TPMs, the model is constant. Then, we simulated 1000 transcriptional profiles for each cell type by randomly sampling from a negative binomial distribution with their corresponding zinb-wave model estimated µ and θ^2^ parameters and introduced dropouts by sampling from a binomial distribution with the zinb-wave model estimated ^p^. **Supplementary Figure 1** shows how the simulated cells cluster together with the original cells for all cell subtypes in our study.

### Generation of Synthetic Bulk Samples

For simulation of bulk samples, single cell profiles are split into training (65%) and a test sets (35%) (**Tables 3**, **4** and **Supplementary Table 1**). Supplementary files that contain the profile matrix and the metadata of the training and test single cell profiles and the bulk samples generated are provided.

**Table 3:** Numbers of real and simulated cells from breast cancer experiment selected for training and test sets.

CELL TYPES	Train real/sim	Test real/sim	Total Cells real/sim
**Tumor (HER2)**	82/658	57/342	139/1000
**Tumor (Luminal A)**	51/648	24/352	75/1000
**Tumor (Luminal B)**	15/651	10/349	25/1000
**Tumor (TNBC)**	71/643	28/357	99/1000
**STROMA (MIXED CELLS)**	14/650	8/350	22/1000
**CD4TH**	9/653	9/347	18/1000
**CD4TMEM**	5/650	3/350	8/1000
**CD4TREG**	3/651	3/349	6/1000
**CD8**	6/652	6/348	12/1000
**M**	16/651	9/349	25/1000
**PDC**	6/652	6/348	12/1000
**GB**	27/652	18/348	45/1000
**MEMB**	25/647	9/353	34/1000
**TOTAL CELLS**	330/8458	190/4542	520/13000

Cell Types: HER2, LuminalA, LuminalB and TNBC indicates the molecular characterization of the tumor cells. Stroma, contains healthy fibroblasts, endothelial and epithelial cells; CD4Th, CD4+ T helper cells; CD4Tmem, CD4+ T memory cells; CD4Treg, CD4+ T regulatory cells; CD8, CD8+ T cells; M, Monocytes; pDC, plasmacytoid dendritic cells; gB, germinal center B cells; memB, memory B cells.

**Table 4:** Numbers of real and simulated cells from colorectal cancer experiment selected for training and test sets.

CELL TYPES	Train real/sim	Test real/sim	Total Cells real/sim
**CRC**	123/664	59/336	182/1000
**EP**	99/653	54/347	153/1000
**FB**	12/629	10/371	22/1000
**CD4**	7/679	0/321	7/1000
**CD8G-**	8/658	0/342	8/1000
**CD8G+**	10/624	10/376	20/1000
**M**	13/660	4/340	17/1000
**MC**	3/646	2/354	5/1000
**GB**	3/639	3/361	6/1000
**PB**	15/641	5/359	20/1000
**TOTAL CELLS**	293/6493	147/3507	440/10000

Cell Types: CRC: colon epithelial tumor cells. Ep, healthy Epithelial cells; Fb, Fibroblast; CD4, CD4+ T cells; CD8G+, CD8+/Gzmb+ T cells; CD8G-, CD8+/Gzmb- T cells; M, Monocytes; Mc, Macrophages; gB, germinal center B cells; pB, peripheral B cells.

To avoid biases due to the composition of the bulk samples, proportions for the mixtures *(w_1,…,w_k)*, where *k* is the number of cell types available in our sample and ∑*_k_w_k_* = 100, are randomly generated using three different approaches (**Supplementary Figure 2**):

1. Cell proportions are randomly sampled from a truncated uniform distribution with predefined limits according to the *a priori* knowledge (obtained from the single cell analysis itself) of the abundance of each cell type (DataSet 1). A second set is generated by randomly permuting cell type labels on the previous proportions (DataSet2).
2. Cell proportions are randomly sampled as for DataSet1 without replacement (DataSet3). After that, a second set is generated by randomly permuting cell type labels on the previous proportions (DataSet4).
3. Cell proportions are randomly sampled from a Dirichlet distribution (DataSet5).

Bulk samples consist then of the expression level of gene *g* in cell type *c*, i.e. *e_gc_* according to Equation 1:

1 $$e_{gc}=\sum_{i=1}^{w_{i}}e_{gi}$$

### Train DigitalDLSorter DNN Model

We used Deep Neural Networks (Hinton and Salakhutdinov, 2006) to predict the proportions of each cell type in a given bulk RNA-Seq sample.

A fully connected DNN was modelled with keras framework (https://keras.io/) with one input layer of as many inputs as genes detected in the dataset, followed by two hidden layers of 200 neurons each and an output of 10 cell types. RELU was used as the activation function and softmax as output function with ADAM optimizer. Dropout layers were added to reduce overfitting and batch normalization layers to reduce biases. **Supplementary Figure 3A** depicts the scheme.

These parameters were selected after running a grid search (**Supplementary Figure 4**) using the parameter space described in **Supplementary Table S2** over a set of 1200 models that were trained with samples of 2000 bulk profiles from the CRC experiment split into 70/30 training/validation sets.

Among the different loss functions tested, Kullback-Leibler divergence (KLD) was selected. This function is able to keep the different evaluation metrics to a minimum (see **Supplementary Figure 4A**). Models with layers between 128 and 200 neurons produced the best fits with the 200 neurons and 2 hidden layers being the winner (see **Supplementary Figure 4B**).

We set a dropout rate of 0.25 as lower rates have a tendency to over-fit (**Supplementary Figure 4C**). The number of epochs was set to 50 in order to have a good fit but preventing over fitting. Low number of epochs produce models with worse scores and models with epochs over 50 have a tendency to over-fit (**Supplementary Figures 4D, E**).

The model was trained with the simulated bulk samples together with the single cell profiles used for the train set. The order of the samples was randomized in order to avoid single cells to concentrate in one batch. The training was run with 50 epochs and batches of 100 samples. **Supplementary Figure 3B** shows the progression of the accuracy and loss function using the KLD. The loss function was minimized with approximately 30 epochs. Also maximum absolute error, which is highly influenced by the higher proportions, and maximum proportional error, which is more affected by the lower proportions are minimized accordingly (**Supplementary Figure 3B**).

### Cell Type Proportion Estimates of TCGA Samples With DigitalDLSorter and Published Deconvolution Methods

RNA-Seq FPKMs quantification files from TCGA breast and colorectal cancer samples were downloaded from the TCGA Data portal together with their corresponding clinical data. TPMs were calculated from the FPKMs files normalized and filtered for the list of genes used in each model. TPMs matrices were fed into de digitalDLSorter models to obtain estimates of cell type abundances.

For EPIC, MCPCounter and ESTIMATE methods we used standard approaches as described in their respective R packages (https://github.com/GfellerLab/EPIC, https://github.com/ebecht/MCPcounter, https://bioinformatics.mdanderson.org/estimate/index.html). TIMER data was collected from their website (https://cistrome.shinyapps.io/timer/).

### TCGA Survival Analysis

For TCGA survival analysis, the ratio between digitalDLSorter CD8/Monocytes-Macrophage proportions were computed and samples were stratified into those having a CD8/Monocyte-Macrophage ratio over the 90**^th^** percentile and the rest. Analysis from EPIC and MCPCounter estimations were done using their most equivalent ratios (CD8_TCells/Macrophages from EPIC and CD8_T_Cells + Cytotoxic_Lymphocytes/Monocytic_lineage from MCPCounter). Overall Survival during the first 2000 days of follow-up was evaluated using log-rank test between the two groups (>90^th^ CD8/Monocyte-Macrophage ratio vs the rest). Individuals at risk every 500 days are displayed. All statistics and plots were performed using the survival R package.

## Results

### scRNA-Seq Highlights a Different Immune Repertoire in Colorectal and Breast Cancer

**Figure 2:**
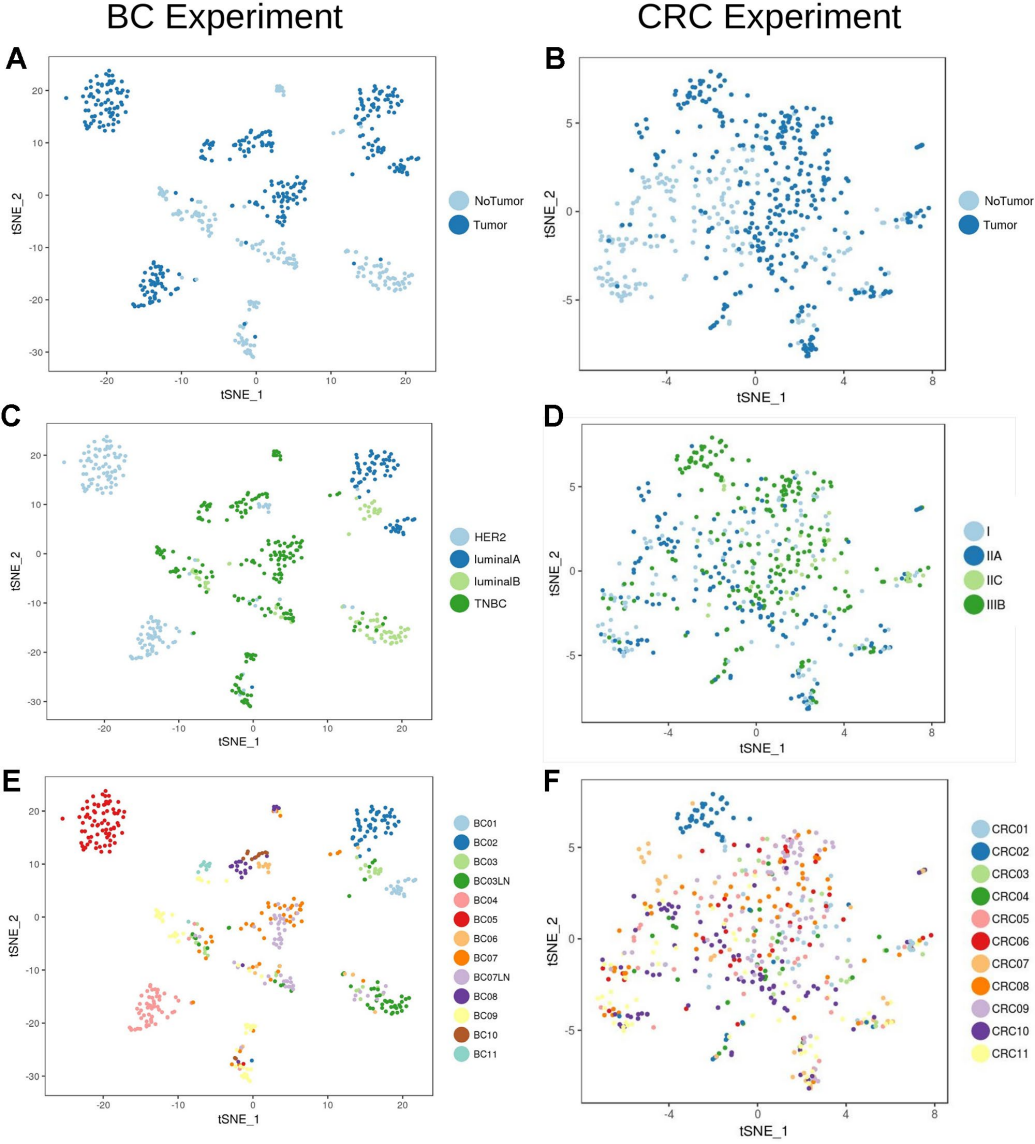
Unbiased t-SNE representation of all single-cells from Breast Cancer and Colorectal Cancer samples. Each dot represent the transcriptome of a single cell. Coloring was done based on: **(A)** Origin (tumor/non-tumor) of BC cells **(B)** Origin (tumor/non-tumor) of CRC cells **(C)** Breast Cancer subtype (ie. HER2, luminal A/B or Triple Negative Breast Cancer, TNBC) **(D)** CRC stage **(E)** BC patient and **(F)** CRC patient.

Analysis of the transcriptional profile of the whole set of cells from BC (**Figures 2A, C, E**) and CRC (**Figures 2B, D, F**) highlights that, for both cancer types, cells clustered based mainly on their origin (tumor/non-tumor, **Figures 2A, B**). Tumor cells from BC samples were very dependent on the tumor subtype (luminal A and B, HER2 and TNBC, **Figure 2C**, **Table 1**). On the contrary, the CRC tumor cells were mixed and difficult to distinguish according to their stage (**Figure 2D**), highlighting a much stronger transcriptional signature per tumor type in breast than in colorectal cancer at the single-cell level.

**Figure 3:**
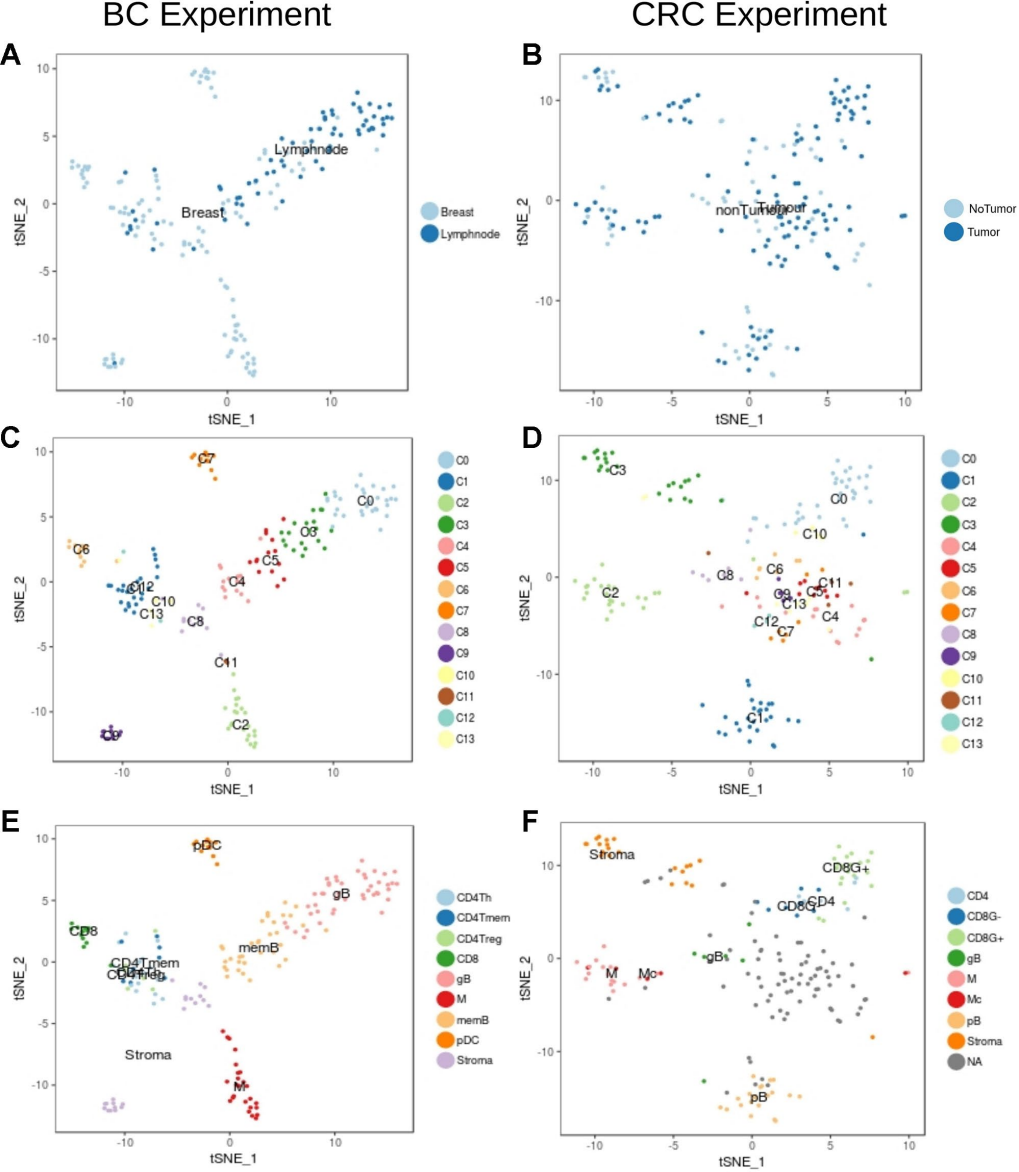
Unbiased t-SNE representation of non-tumor single-cells from Breast and Colorectal samples. Each dot represents a single-cell colored by: **(A)** Non-tumor cells extracted from the BC tumor sample or from matched lymph nodes **(B)** Non-tumor cells from the CRC tumor sample or from matched healthy tissue **(C)** Graph-based clusters identified by Seurat in the BC experiment and in the **(D)** CRC. **(E)** and **(F)** colored by the cell type classification.

We then focused in the analysis of non-tumor cells to understand the transcriptional characteristics of the immune infiltration in the different tumor types. Graph-based clustering followed by t-sne visualization of the non-tumor cells (**Figure 3**) identified several clusters of immune cells within the BC (**Figure 3C**) and the CRC (**Figure 3D**) experiments. These clusters were then characterized based on the results from xCell software (**Supplementary Figures 5** and **6**) and on the manual inspection of markers (**Supplementary Figures 7**–**12**).

Within the BC non-tumor cells we identified several clusters of T cells (clusters C1, C6, C10, C12 and C13, **Figure 3C**) characterized by high levels of CD3 genes and TCRs (**Supplementary Figure 7**). Among them, cells in cluster C6 (**Figure 3C**) were labelled as CD8+ due to the expression of high levels of CD8A gene and GZMB, indicative of its activity status (**Supplementary Figure 7**). CD4+ cells were spread across clusters C1, C10, C12 and C13. Based on xCell results and according to the expression of CD4 (**Supplementary Figure 7**) we identified three different subpopulations: CD4Tm, CD4Th and CD4Treg (**Figure 3E** and **Supplementary Figure 6**). Finally, B cells (clusters C0, C3, C4 and C5, **Figure 3C**) were identified based on the expression of CD19, CD20 and Immunoglobulins (IG) (**Figure 3**, **Supplementary Figures 6** and **8**) (Shen et al., 2004). In particular, clusters C0 and C3 contained proliferative germinal center B cells (gB) with high levels of proliferative markers like CR2 and MKI67 (**Supplementary Figure 8**) while clusters C4 and C5 contain more mature memory B cells (memB), with higher levels of IGHM and CD27 (**Supplementary Figure 8**).Clusters C2 and C11 were considered monocyte derived based on CD14, PTPRC and ITGAX expression (Collin and Bigley, 2018) (**Figure 3**, **Supplementary Figures 6** and **9**). However, it was not possible to clearly identify the macrophages within this cluster based on our tools. Finally, xCell classified cells in the cluster C7 as plasmacytoid DCs (pDC). The remaining cells (clusters C8 and C9) were classified by xCell as fibroblasts and endothelial cells and we considered them Stroma.

In the set of CRC non-tumor cells, clusters C0 and C10 contained cells classified as T-cells based on the expression of CD3 genes (**Figures 3B, D, F**, **Supplementary Figures 5** and **10**). The separation of CD8 and CD4 cells was however not clear due to the low levels of expression of CD8 and CD4 markers and its large dispersion in these cells. Hence, the T cell population on cluster C0 was finally divided into GZMB+ and GZMB- cells (**Supplementary Figure 10**). Among GZMB- cells some were identified as CD4+ according to xCell (see **Figure 3**, **Supplementary Figures 5** and **11**). Although CD19 was not captured properly in this experiment, other markers like IGs, CD27 and the xCell classification defined cells in clusters C1 and C8 as B cells. C1 contains mature B cells (pB) while cluster C8 contains more proliferative B cells which probably belong to tumor associated germinal centers (gB) (**Figure 3**, **Supplementary Figures 5** and **11**). Monocytes were identified in cluster C2. Unlike in the BC set, xCell identified a few macrophages in cluster C2 (see **Figure 3**, **Supplementary Figures 5** and **12**). As in BC, clusters C3 and C13 had a mix of fibroblasts, smooth muscle cells and endothelial cells that were all considered as Stroma (see **Figure 3**, **Supplementary Figure 4**). Tshe rest of the clusters had a mixed transcriptome difficult to interpret and were hence not considered for posterior analysis (NAs in **Figure 3F**).

Overall, the differences in the types of cells identified in both experiments highlights the importance of the context in the nature of the cell profiles and the necessity to define tumor specific cell profiles for the correct enumeration of cell proportions form bulk RNA-Seq samples.

### DigitalDLSorter Predicts the Proportion of Each Cell Type in Bulk RNA-Seq Samples

**Figure 4:**
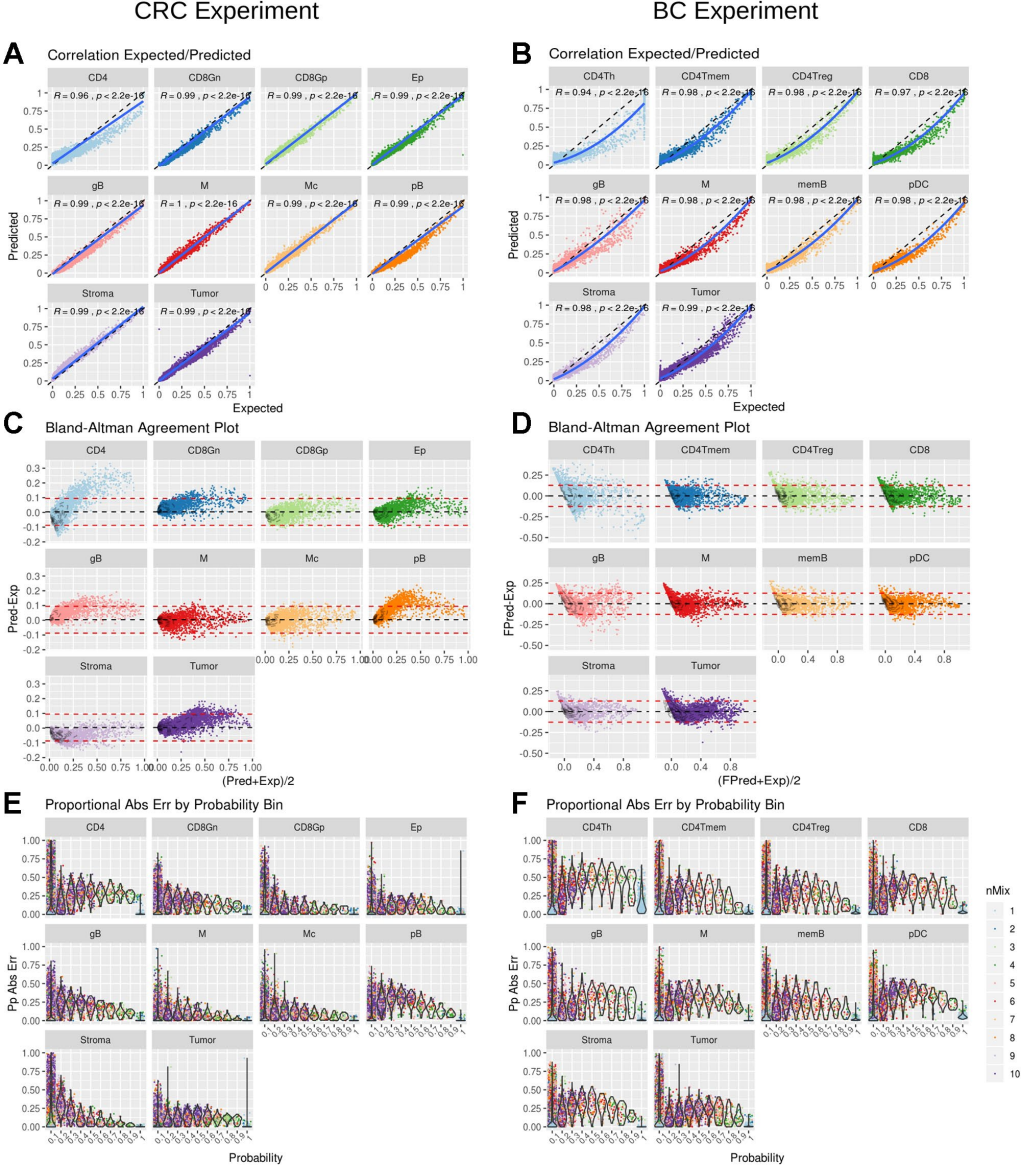
DigitalDLSorter performance in the test set. Correlation between real (Expected) and Predicted proportions for each cell type from the CRC model **(A)** and BC model **(B)**. Correlation coefficient and p-value of a linear (CRC) and quadratic fit (BC) are shown. The solid line represent the fit of the corresponding model. The dashed line represents the identity. Bland-Altman agreement plot for the CRC **(C)** and the BC **(D)** models. The red dashed lines represent +/- 1.96 x std deviance from the mean (dashed black line) **(E)** and **(F)** Absolute Proportional Error binned by frequency and arranged by cell type for CRC **(E)** and BC **(F)** models.

DigitalDLSorter estimates the proportion of each cell type present in a given bulk sample. Hence, its performance was assessed based on the correlation and the agreement of the predicted proportions versus the actual ones for the test set, based on the model obtained from the training data (**Figure 4**). For CRC there was a linear relationship between the predicted and the real data with a correlation of 0.99 (p < 0.001), except for CD4 cells (**Figure 4A**), for which actual proportions between 25% and 75% were slightly underestimated, with yet a good overall correlation of 0.94 (p < 0.001). Agreement was also very good for all cell types except for CD4 using a Bland-Altman plot (**Figure 4C**). Interestingly, BC predictions were also in good agreement with the actual proportions although the relation between them was not lineal but quadratic (**Figure 4B**). Square root transformation and scaling of the predicted proportions (**Supplementary Figure 13**) showed a good agreement with the original proportions (**Figure 4D**). The same effect was also observed when looking at the maximum proportional error, which is in general larger for the lower proportion bin in the BC model (**Figure 4F**) than in the CRC model (**Figure 4E**).

### DigitalDLSorter Predictions Are Biologically Well Sustain and Correlate With Those From Other Deconvolution Tools

The model obtained from digitalDLSorter was then applied to quantify the proportion of the different immune and non-immune cell subtypes in bulk RNA-Seq samples from breast (n = 1208) (Koboldt et al., 2012; Ciriello et al., 2015) and colorectal cancer patients (n = 521) (Atlas et al., 2012) from TCGA.

**Figure 5:**
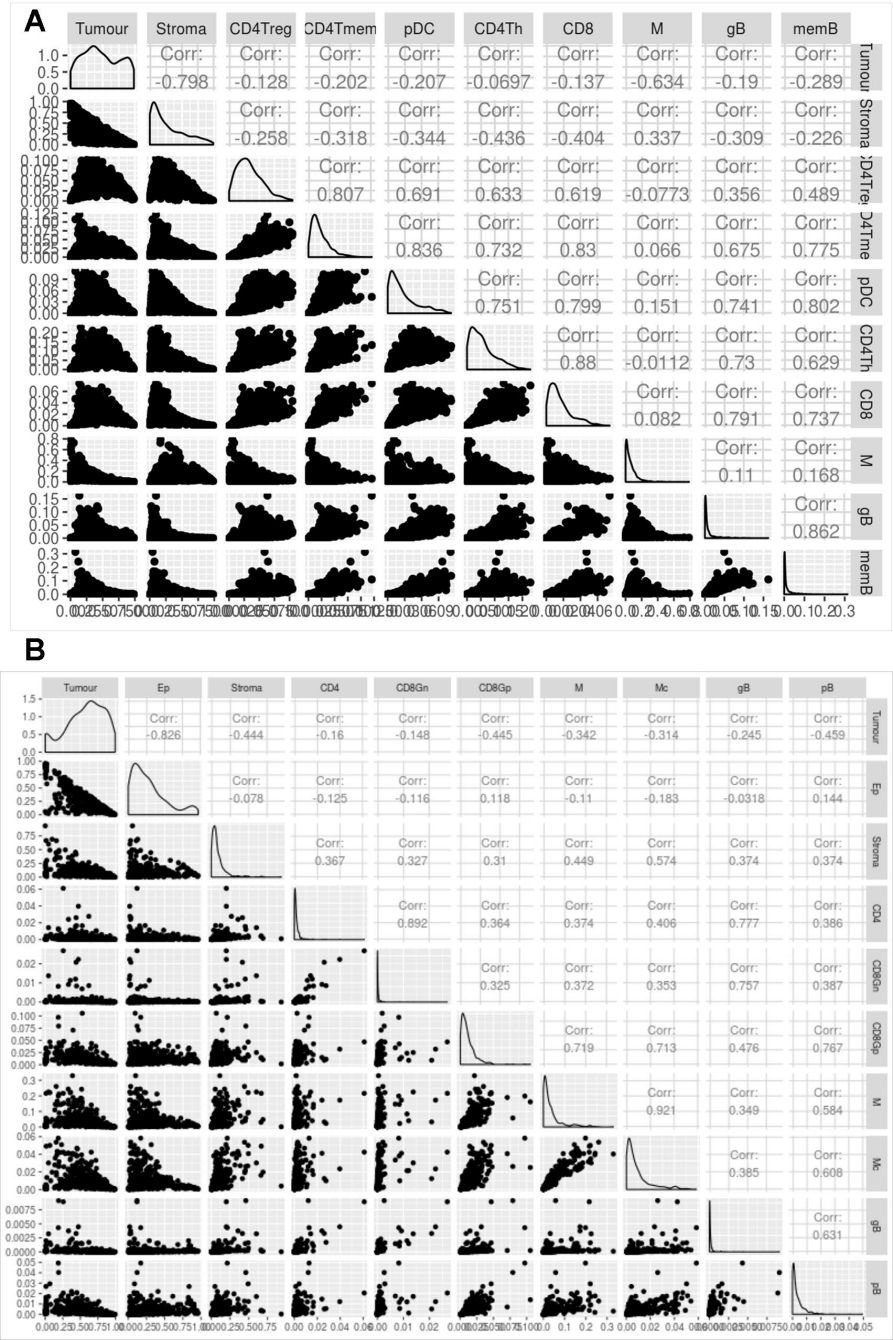
Evaluation of digitalDLSorter predictions in TCGA samples. Correlation matrix of the different cell types evaluated by the digitalDLSorter model for BC **(A)** and CRC **(B)**. Correlation coefficients are shown for all pairwise comparisons.

**Figure 5** shows the correlation between the proportions of the different cell types predicted by digitalDLSorter across samples for BC (**Figure 5A**) and CRC (**Figure 5B**) models. The anticorrelation (r∼-0.8) of tumor and stroma (Epithelial and Stroma cells for CRC) proportions in both models is a good indication of the good performance of digitalDLSorter. Additionally, tumor and stroma anticorrelate with most of the immune cell types. In BC cells, we found a strong positive correlation (>0.7) between B and T cells (CD4Th, CD4Tm and CD8+). In contrast, as expected, CD4Treg had a poor correlation with CD8 or CD4Th, confirming the validity of digitalDLSorter estimates.

**Figure 6:**
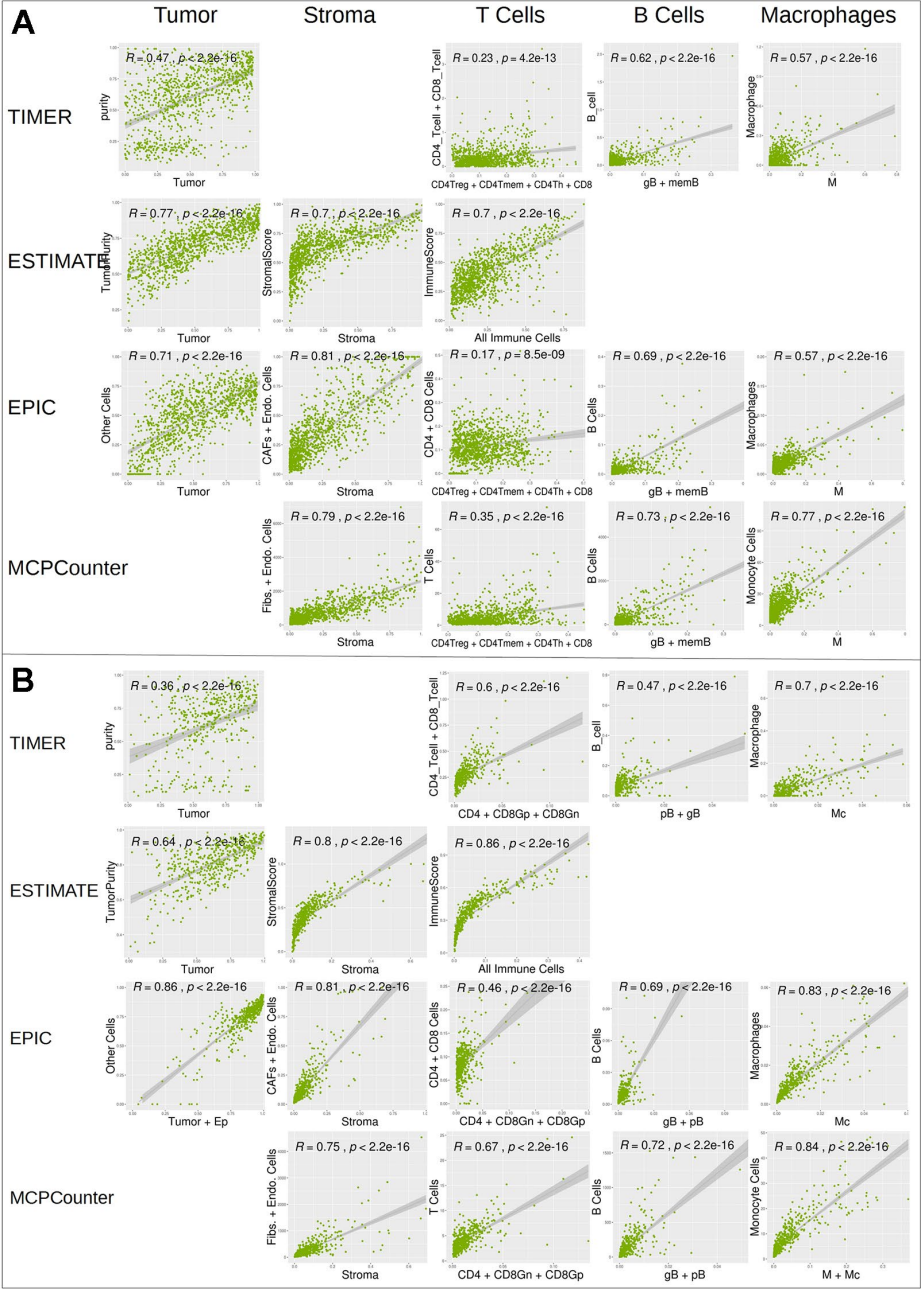
Correlation of digitalDLSorter predictions with TIMER, ESTIMATE, EPIC and MCPCounter tools. Correlation of the cell type content estimated from digitalDLSorter (x-axis) and estimates from TIMER, ESTIMATE, EPIC and MCPCounter (y-axis) for BC **(A)** and CRC **(B)** TCGA samples. EPIC Other Cells (which represents the content of all cells, malignant or not, not present in its set of cell type signatures) is correlated with digitalDLSorter tumor **(A)** or tumor+Ep cells **(B)**. ImmuneScore from ESTIMATE (calculated from a gene signature that contains signals for T cells, B cells and APC cells) is correlated with the combination of all immune cells from digitalDLSorter model and is placed under the T Cells column for convenience (All Immune Cells = CD4Th+CD4Tmem+CD4Treg+CD8+gB+memB+M+pDC for BC model or CD4+CD8Gn+CD8Gp+pB+gB+M+Mc for CRC model). digitalDLSorter Stroma (Stroma column) is compared with a combination of Fibroblasts and Endothelial cells for EPIC and MCPCounter estimations and with the StromalScore for ESTIMATE. digitalDLSorter combinations of all CD4 and CD8 T cell types (T Cells column) is compared with the corresponding combinations of the other tools (CD4 + CD8 for TIMER, CD4+CD8 for EPIC and T Cells for MCPCounter). digitalDLSorter combination of all B Cell types (B Cells column) and Macrophages plus Monocytes (Monocyte column) are compared with those from TIMER, EPIC and MCPCounter.

We further compared digitalDLSorter estimations with those from TIMER, ESTIMATE, EPIC and MCPCounter (**Figure 6**). Given the different cell type models produced by each tool, we combined the cell type proportions into 5 categories: Tumor, Stroma (combination of Fibroblasts and Endothelial cells), T Cells (combination of all CD3 cell types), B cells (combination of all B cell types) and Monocytes (combination of Monocytes and Macrophages).

DigitalDLSorter tumor content (first column on **Figures 6A, B**) shows very good correlations with tumor content estimations form EPIC, ESTIMATE and TIMER. digitalDLSorter tumor content shows the lowest correlations with TIMER purity probably due to the presence of a bimodal distribution in the TIMER purity estimations and the different nature of the purity value in TIMER (DNA based and on a different portion of the biopsy) (Li and Li, 2014). Most importantly, digitalDLSorter shows very similar predictions with the other models especially in Stroma, B cells and Monocyte lineages and in the CRC model (**Figure 6**).

**Figure 7:**
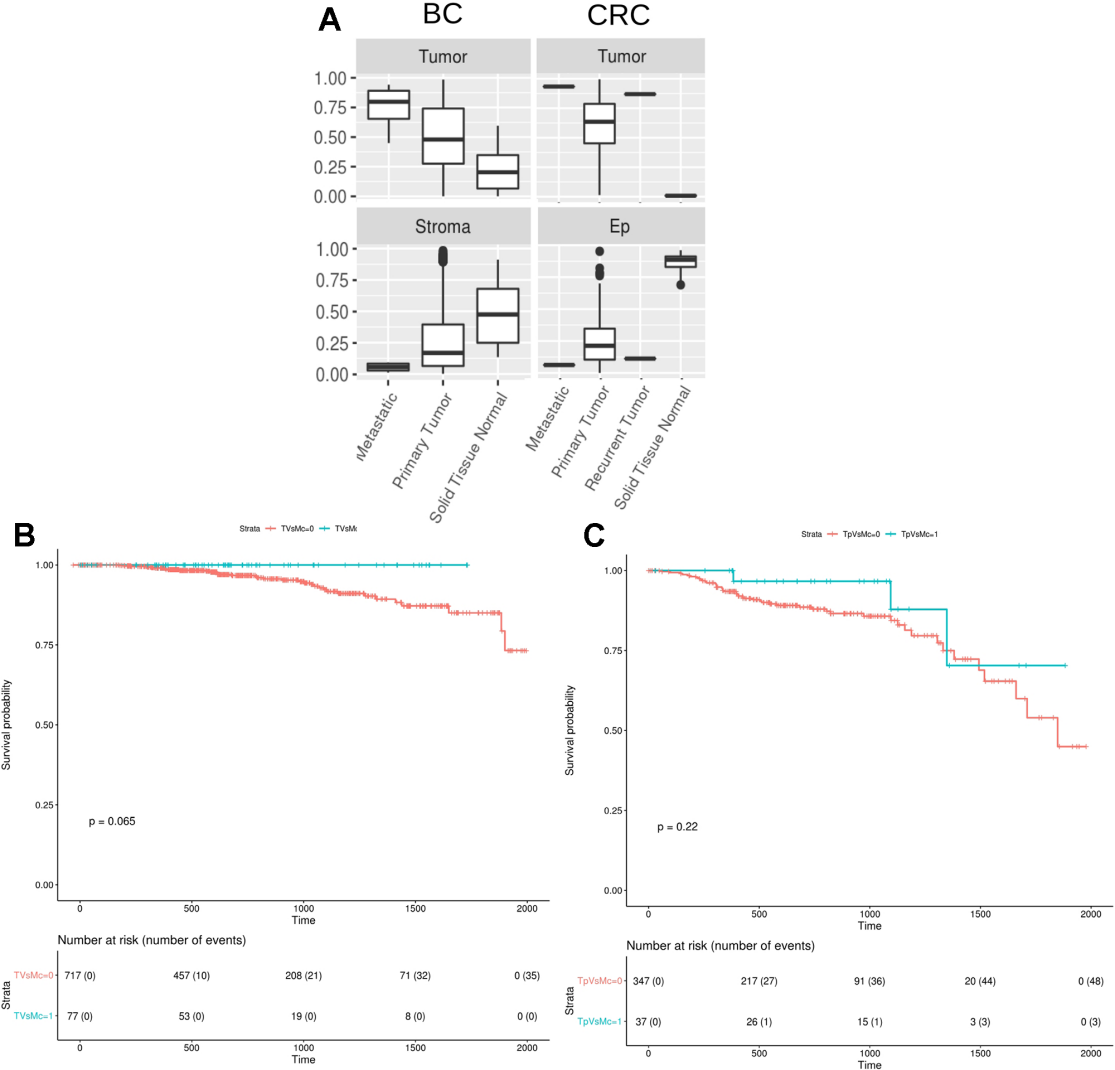
DigitalDLSorter estimations of the tumor immune infiltration is predictive of the overall survival of Breast and Colorectal Cancer patients. **(A)** Tumor and Stroma or Ep cells abundance from BC (left) and CRC (right) TCGA samples grouped by sample type (metastatic, primary tumor, recurrent tumor, normal tissue). **(B, C)** Kaplan-Meier overall survival curves from breast **(B)** and colorectal **(C)** cancer patients. In blue, samples within the highest 90^th^ quantile of the ratio between T cells (CD8+CD4Th+CD4Tmem for BC, CD8Gp for CRC) over Monocytes/Macrophages (Mono). In red, individuals with low Tcells/Mono ratio.

Finally, we studied the digitalDLsorter-estimated proportion of tumor cells in the TCGA samples grouped by the type of biopsy, *i.e. primary tumor, paired normal tissue, recurrent tumor* or *metastatic sample* (**Figure 7A**). According to what it would be expected, DigitalDLSorter predicts low levels of tumor cells in normal tissues, especially for the CRC samples, and higher levels for recurrent and metastatic samples, reinforcing the validity of our model.

### The Amount and Type of Immune Infiltration Estimated With DigitalDLSorter Predicts Survival of TCGA Breast and Colorectal Cancer Patients

Tumor infiltrated lymphocytes (TILs) and especially T cells have been extensively reported as predictors of good prognosis for overall and disease-free survival on different types of cancers (Galon et al., 2006). On the contrary, macrophages have been reported to have protumoral activity (Bingle et al., 2002). Based on the digitalDLSorter estimations of CD8 and Monocytes-Macrophages (MM) proportions from bulk RNA-Seq data, we assessed the survival of TCGA individuals based on their CD8+/MM ratio. Patients with a high CD8+/MM ratio had a better survival in both cancer types (**Figure 7B**), versus those individuals with a lower CD8+/MM ratio. In spite of this interesting result, significance was not achieved probably due to the small number of individuals in the group with high ratios (p = 0.06 for BC and p = 0.22 for CRC). None of the other models did produce better stratification of the patients survival based on the CD8/MM ratio (**Supplementary Figure 14**). These results support the validity of the estimations produced by digitalDLSorter.

## Discussion

The explosion in the use of scRNA-Seq that we are currently experiencing evidences the long suspected idea that the heterogeneity of cells at the transcriptional level expands beyond a handful of markers, as previously believed. It also completely changes the paradigm about the information extracted from differential gene expression studies using bulk RNA samples. In particular, the field of immunology is being revolution by this technique, allowing detailed study of the heterogeneity of the immune response in different diseases ranging from cancer to atherosclerosis.

In spite of its constantly decreasing cost, scRNA-Seq is still expensive. For that reason and in light of the findings produced in the last years thanks to this technology deconvolution methods are becoming the preferred tool for the analysis of bulk RNA-Seq in tissues with a large cellular heterogeneity.

In spite of its cost, a single experiment of scRNA-Seq holds such a vast amount of information that published data are often re-analyzed and integrated with other data to answer questions different from that for which they were generated. Analysis of scRNA-Seq is challenging, requiring a reasonable amount of knowledge about the biological question of interest. However, its strength lies in the large number of cells (individuals) available, making it a perfect type of data for the application of machine learning methods (Lopez et al., 2018; Way and Greene, 2018) that were up to date of limited help in molecular research.

In this paper we present a new method for the deconvolution of bulk RNA-Seq data using scRNA-Seq profiles. Taking advantage of the large dimensionality of this type of data we used a deep neural network to train a model based on synthetic mixtures of single cell populations. To exemplify our method, we used two previously published datasets about breast and colorectal cancer. These single cell datasets, although small, they have provided enough information to dissect at least 10 cell types and to train DNN models, which did reflect the cellular interplay between the different cell types in the different tumor context. DigitalDLSorter models also highlights the importance of producing deconvolution models tailored for each tumor and represents a straight forward methodology to produce those models from specific single cell experiments.

The digitalDLSorter deconvolution models may benefit from deeper single cell experiments as low frequent cell types will become more evident and dissectible. We chose these two experiments because they used similar technology and were easier to compare, but more importantly because they did not go through a sorting process that could modify the cell types obtained. Many single cell experiments carried to date on tumor samples have been subject to a process of filtering that may exclude unexpected immune cell types important for the tumor etiology, besides of excluding non-immune cells. To generate good models is imperative to start from unbiased single cell experiments. An ideal experiment would have 40000 cells from 10 to 20 patients which would provide a detailed source of information to build an accurate digitalDLSorter model.

We are currently expanding our repository of cell types to be able to use our deconvolution method in different scenarios for which immune cells are important. We believe that our method can be of great used to the community to extract information about the immune cells.

## Data Availability Statement

We analyzed two single cell RNASeq experiments from the literature and stored in the Gene Expression Omnibus database (GEO: https://www.ncbi.nlm.nih.gov/geo/ – GSE75688 and GSE81861).

## Ethics Statement

This study was carried with human open access data from with their corresponding ethics committee approval.

## Author Contributions

FS-C conceived the study, CT implemented all analysis and produced the figures. CT and FS-C wrote the manuscript.

## Funding

The results shown here are in part based upon data generated by the TCGA Research Network: https://www.cancer.gov/tcga. This work was supported by the European Union’s Horizon 2020 research and innovation program under grant agreement number 633592 (Project APERIM: Advanced bioinformatics platform for personalized cancer immunotherapy) and by the Ministerio de Ciencia, Innovación, y Universidades (MCIU) [grant no. RTI2018-102084-B-I00]. The CNIC is supported by MEIC and the Pro CNIC Foundation, and is a Severo Ochoa Center of Excellence (MEIC award SEV-2015-0505).

## Conflict of Interest

The authors declare that the research was conducted in the absence of any commercial or financial relationships that could be construed as a potential conflict of interest.

## Abbreviations

DL, Deep Learning; scRNA-Seq, single-cell RNA-Seq; CD8, CD8+ Citotoxic T Cells; CD4, CD4+ Helper T Cells; NK, Natural Killer Cells; Ig, Immunoglobulins; BC, Breast Cancer; CRC, Colorectal Cancer; DNN, Deep Neural Network; TCGA, The Cancer Genome Atlas; TPM, Transcripts per Million; FPKM, Fragments per Transcript Kilobase per Million Reads; GEO, Gene Expression Omnibus; CD4Th, CD4+ Helper T Cells; CD4Tm, CD4+ Helper memory T Cells; CD4Treg, CD4+ Regulatory Helper T Cell; SC, Single Cell; KLD, Kullback-Leibler Divergence; TIL, Tumor Infiltrating Lymphocite; ML, Machine Learning; M, Monocytes; Mc, Macrophages; CNV, Copy Number Variation.
