# Corrigendum: Digitaldlsorter: Deep-Learning on scRNA-Seq to Deconvolute Gene Expression Data

**DOI:** 10.3389/fgene.2019.01373

**Published:** 2020-02-06

**Authors:** Carlos Torroja, Fatima Sanchez-Cabo

**Affiliations:** Bioinformatics Unit, Fundación Centro Nacional de Investigaciones Cardiovasculares (CNIC), Madrid, Spain

**Keywords:** machine learning, deconvolution algorithm, cancer, immunology, single-cell

In the original article, the funder “Ministerio de Ciencia, Innovación, y Universidades (MCIU), RTI2018-102084-B-I00” to “Fatima Sanchez-Cabo” was missing. The corrected Funding Statement follows below:

“The results shown here are in part based upon data generated by the TCGA Research Network: https://www.cancer.gov/tcga. This work was supported by the European Union’s Horizon 2020 research and innovation program under grant agreement number 633592 (Project APERIM: Advanced bioinformatics platform for personalized cancer immunotherapy) and by the Ministerio de Ciencia, Innovación, y Universidades (MCIU) [grant no. RTI2018-102084-B-I00]. The CNIC is supported by MCIU and the Pro-CNIC Foundation and is a Severo Ochoa Center of Excellence [MCIU award SEV-2015-0505].”

Additionally, the **Ethics Statement**, **Author Contributions** and **Acknowledgements** statement were not included in the original Article. The Statements follows below:

Ethics Statement:

“This study was carried with human open access data from with their corresponding ethics committee approval.”

Author Contributions:

“FS-C conceived the study, CT implemented all analysis and produced the figures. CT and FS-C wrote the manuscript.”

Acknowledgements:

“We would like to thank Francesca Finotello and Zlatko Trajanoski for fruitful discussions and to the CNIC Bioinformatics Unit members for continuous support and work.”

The authors apologize for these errors and state that this does not change the scientific conclusions of the article in any way. The original article has been updated.

