# Supplementary material for: Digitaldlsorter: Deep-Learning on scRNA-Seq to Deconvolute Gene Expression Data
Source: Front Genet. 2019 Oct 25;10:978. doi: 10.3389/fgene.2019.00978 (PMC6824295; doi:10.3389/fgene.2019.00978)
Supplement: Supplementary file 1 [file DataSheet_1.pdf]

Supp Table 1: Number of Synthetic bulk mixtures generated in each dataset.

DATASET	TRAIN	TEST
**DATASET1**	1000	700
**DATASET2**	2000	1400
**DATASET3**	1000	350
**DATASET4**	3500	2700
**DATASET5**	3000	2000

Supp Table 2: Grid Search Parameter space. Range of values randomly sampled for each parameter for models generation during grid search analysis.

PARAMETER	RANGE
**LR**	0.01-1
**NEURONS**	32-256
**HIDDEN_LAYERS**	0-2
**BATCH_SIZE**	10-500
**EPOCH**	10-100
**DROPOUT**	0-0.4
**OPTIMIZER**	adam, nadam
**LOSS**	mse, mean_absolute_percentage_error, mean_abosulte_error, kullback_leibler_divergence
**ACTIVATION**	relu
**OUTPUT_ACTIVATION**	softmax
**KERNEL_INITIALIZER**	normal, uniform
**BATCH_NORMALIZATION**	True, False
**NUMBER OF CLASSES**	10

Supp. Figure 1.

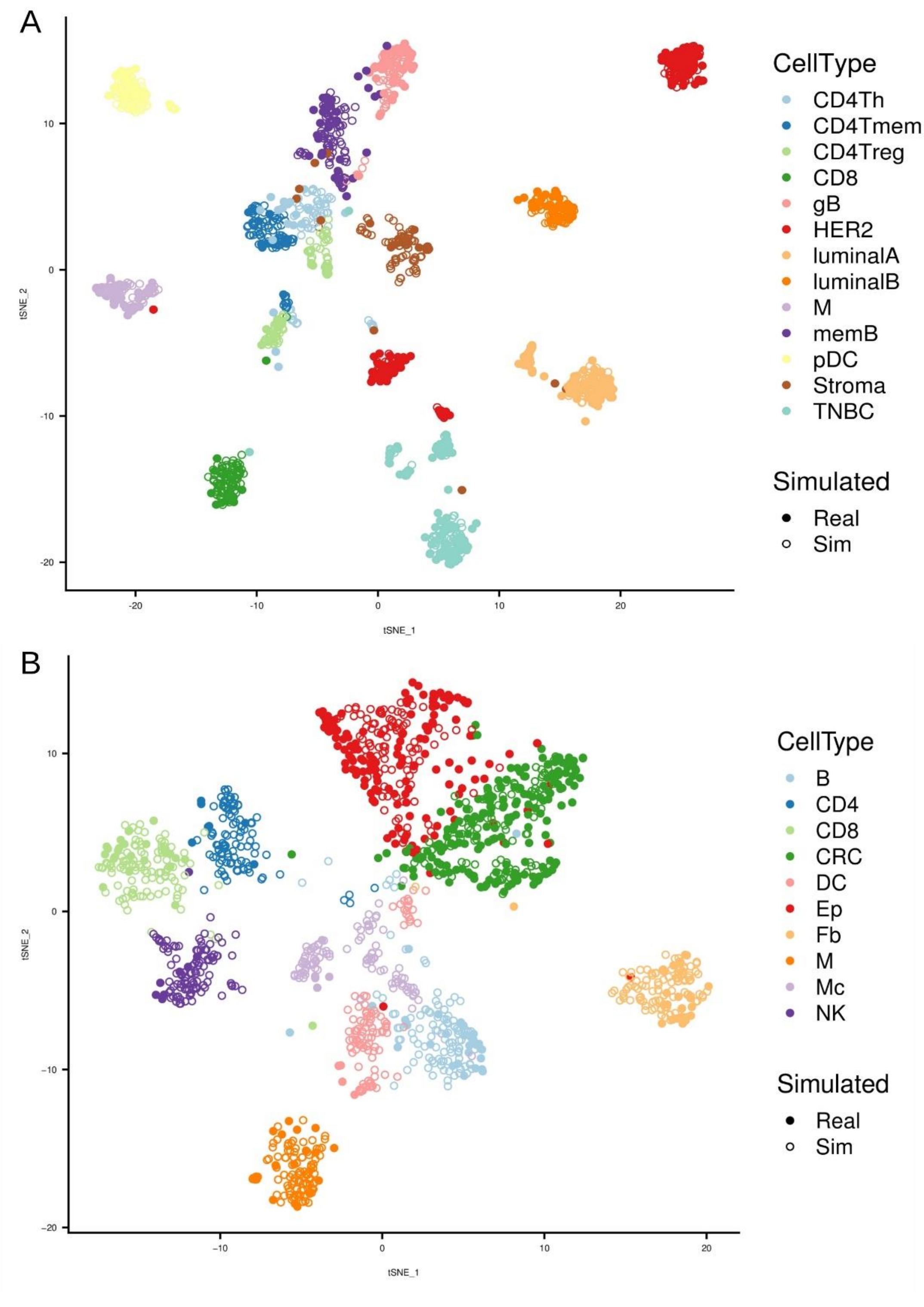

Supp. Figure 2.

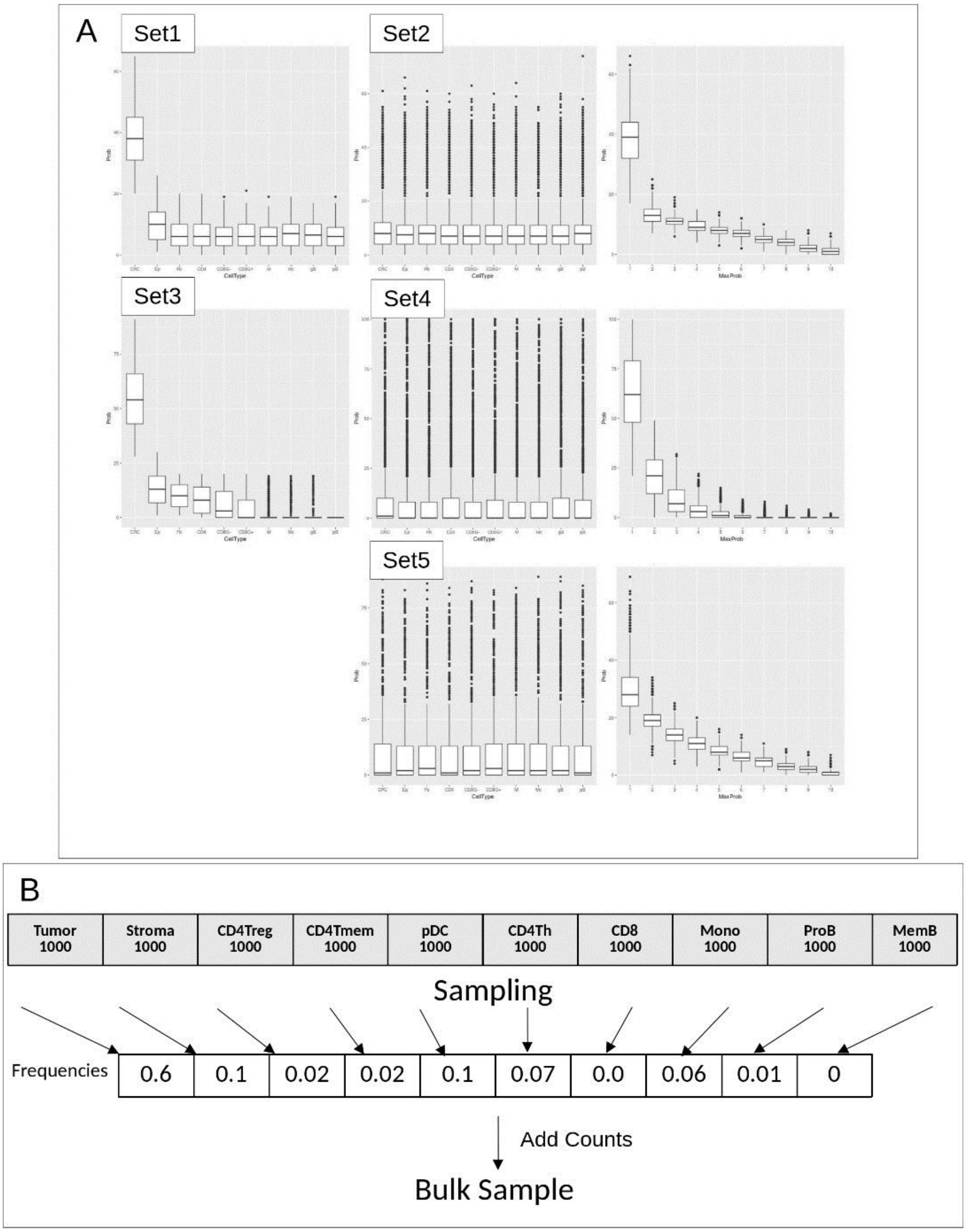

Supp. Figure 3.

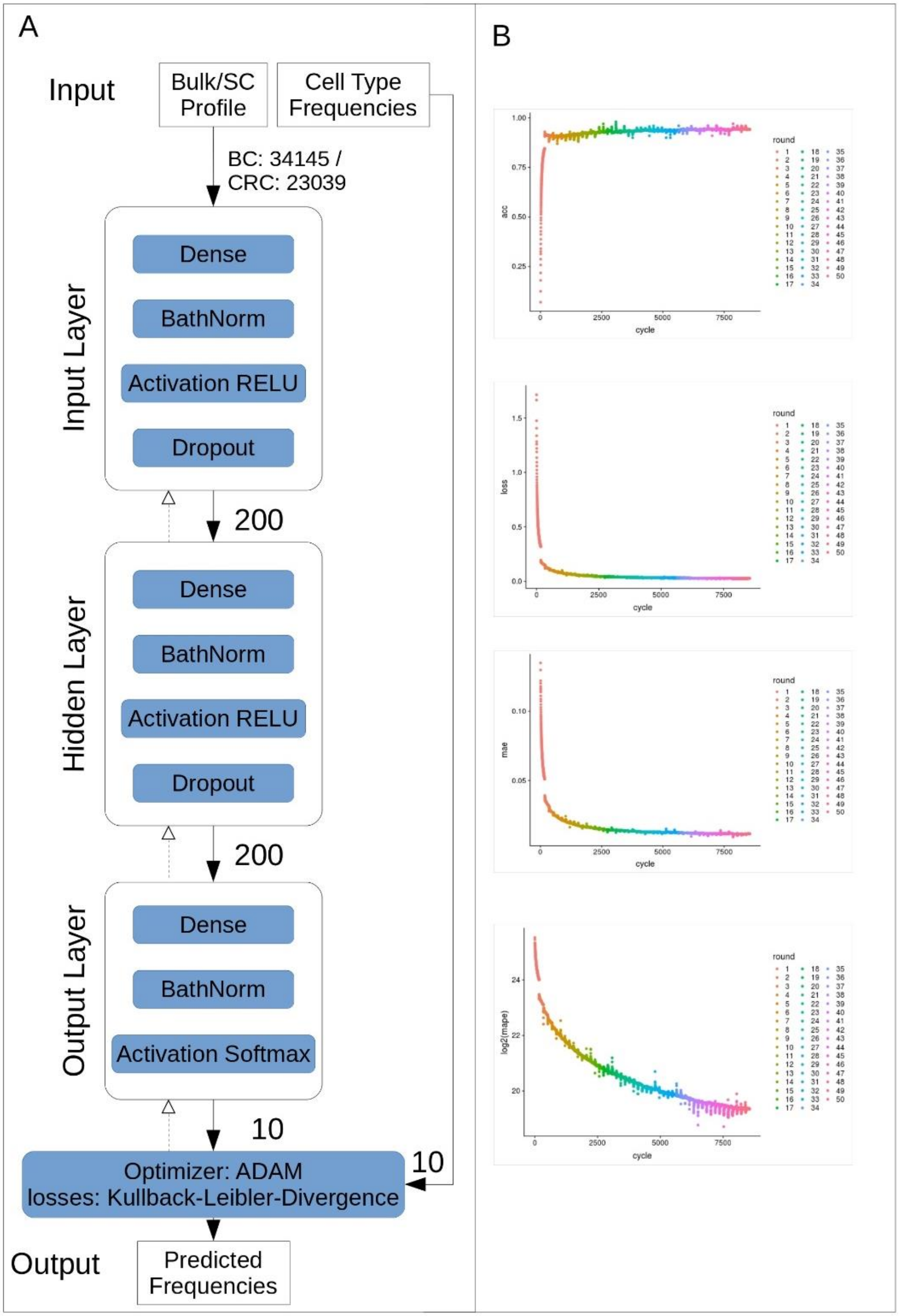

Supp. Figure 4.

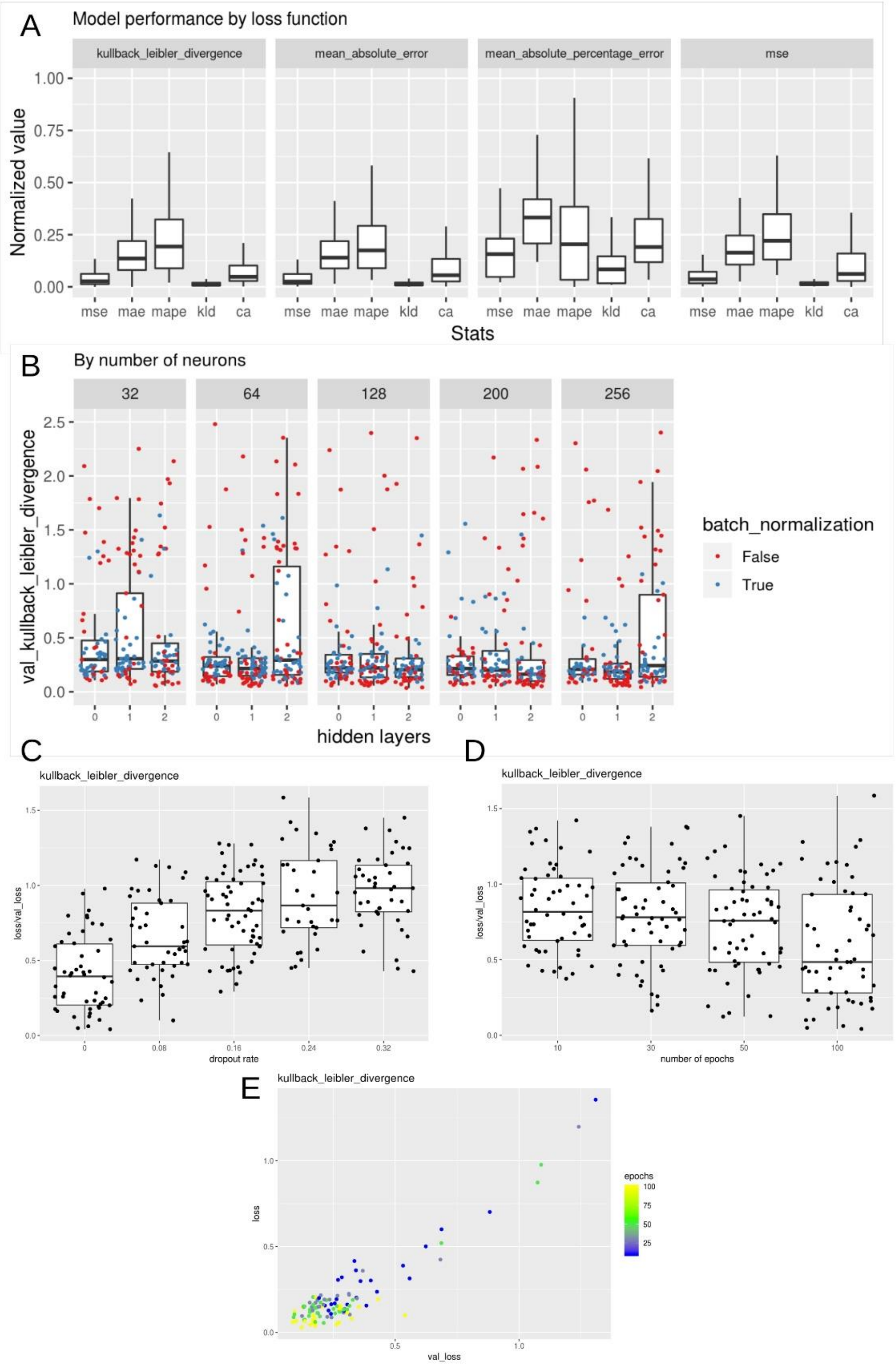

Supp.Figure 5.

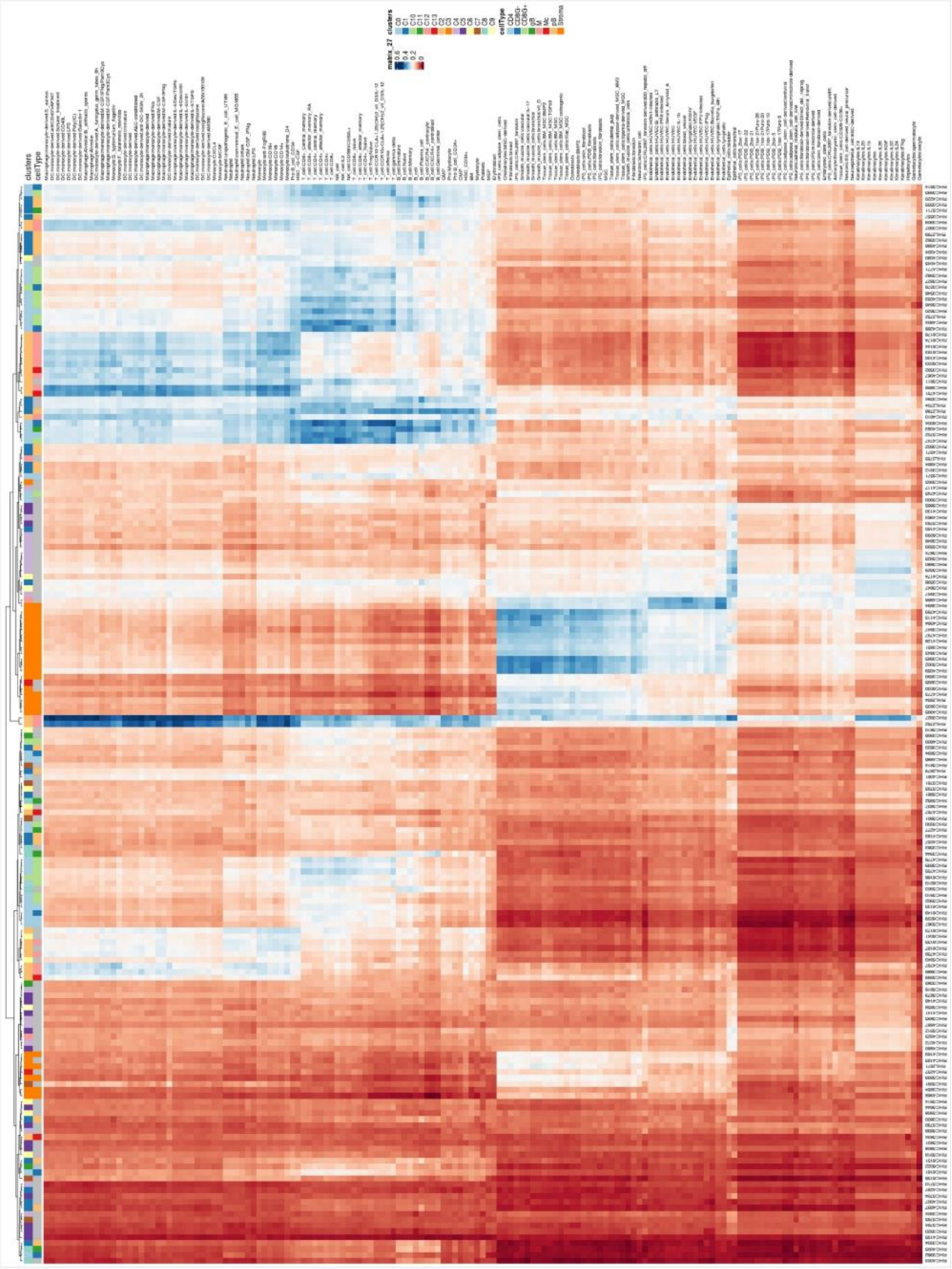

Supp.Figure 6.

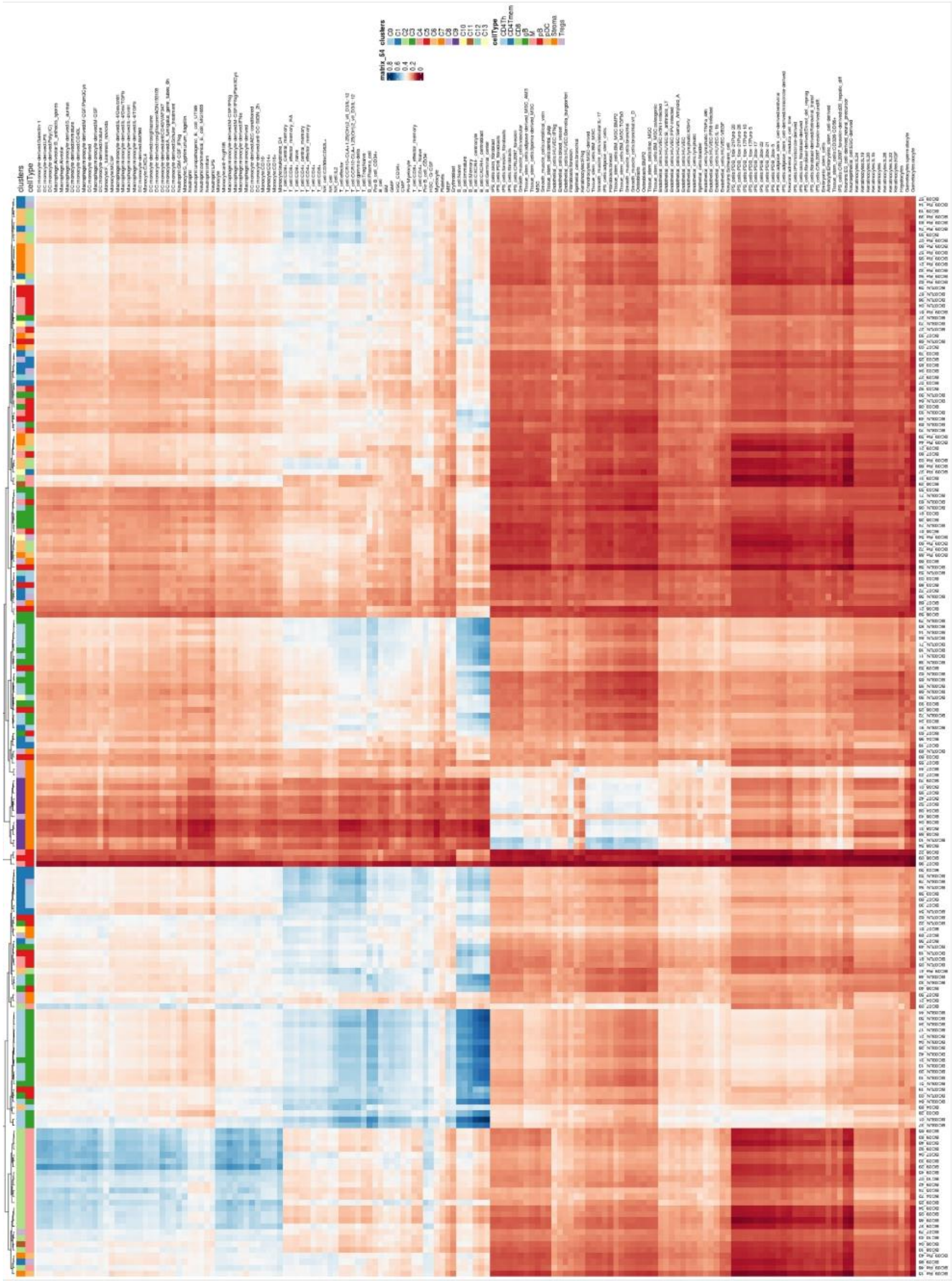

Supp. Figure 7.

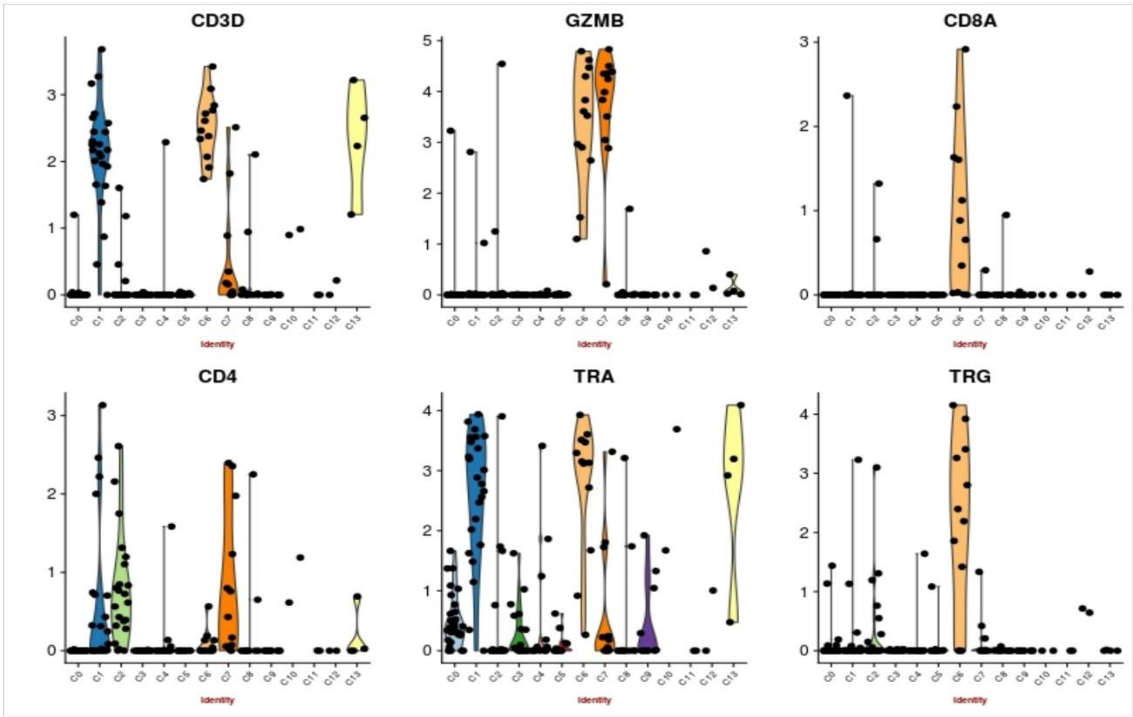

Supp. Figure 8.

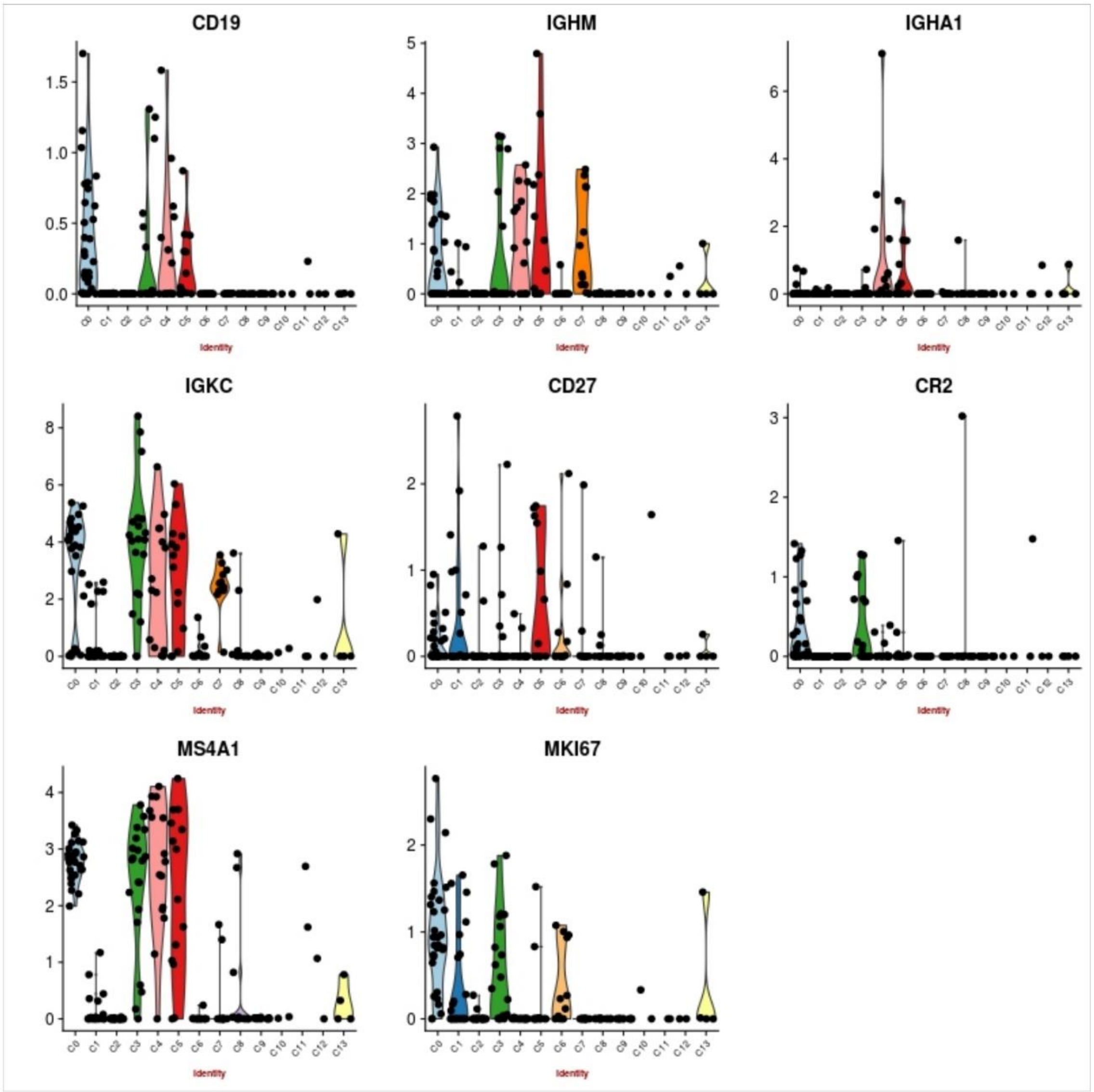

Supp. Figure 9.

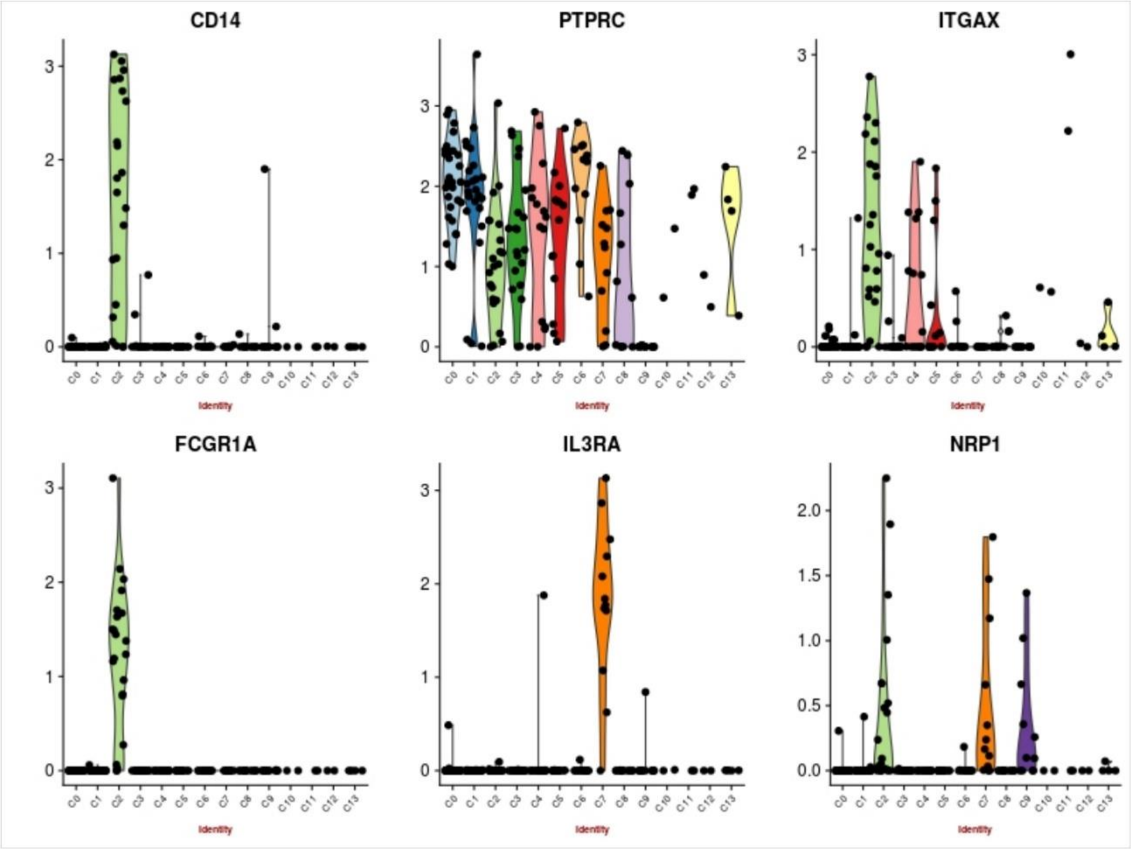

Supp. Figure 10.

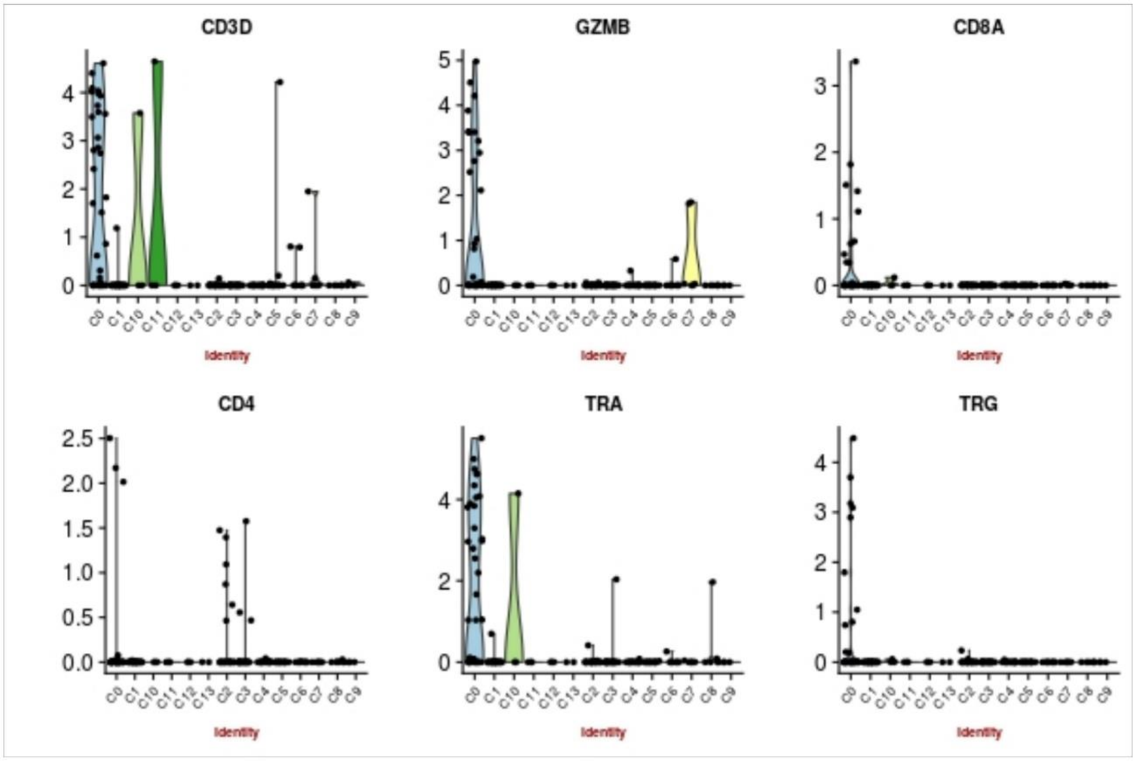

Supp. Figure 11.

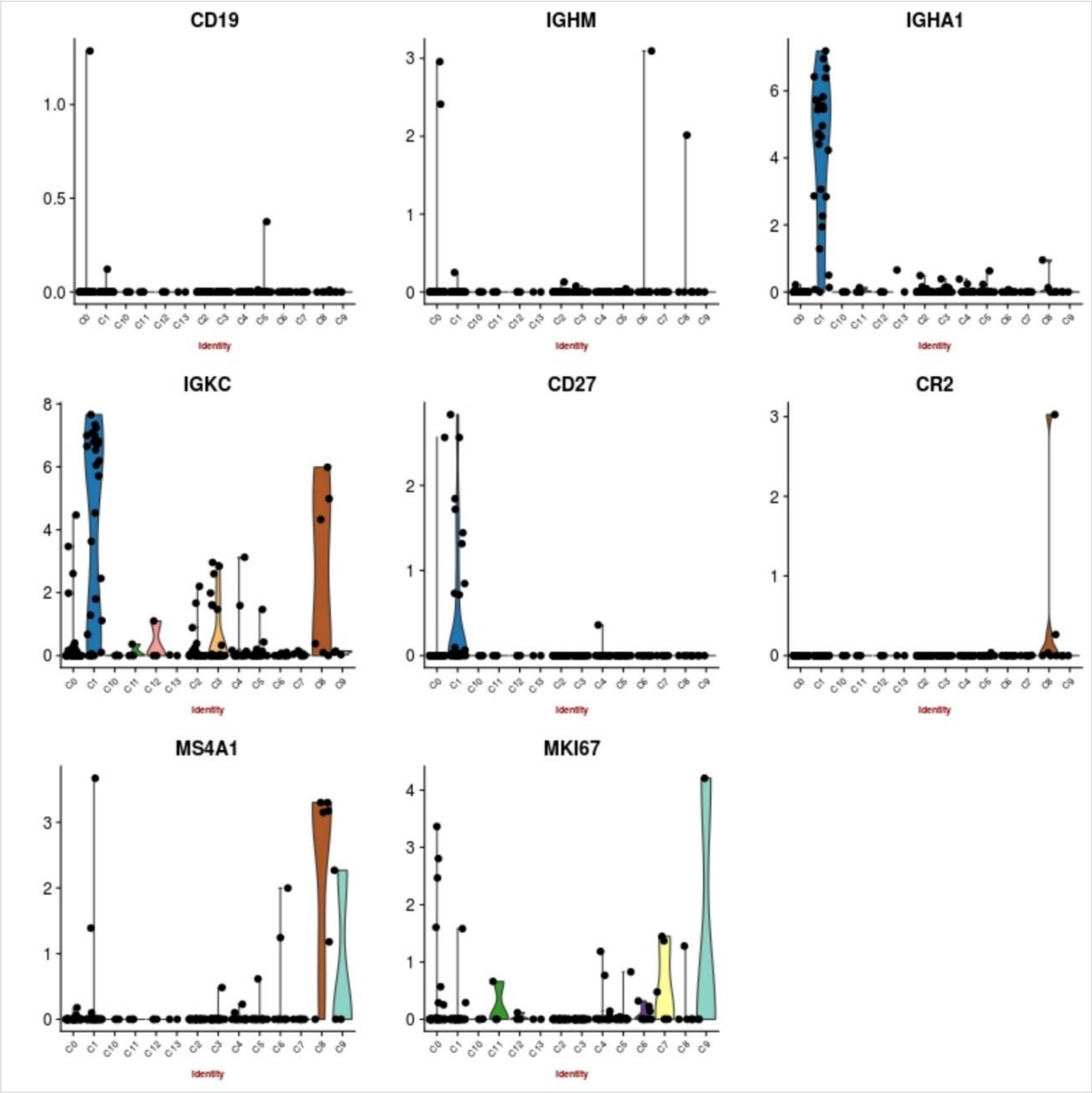

Supp. Figure 12.

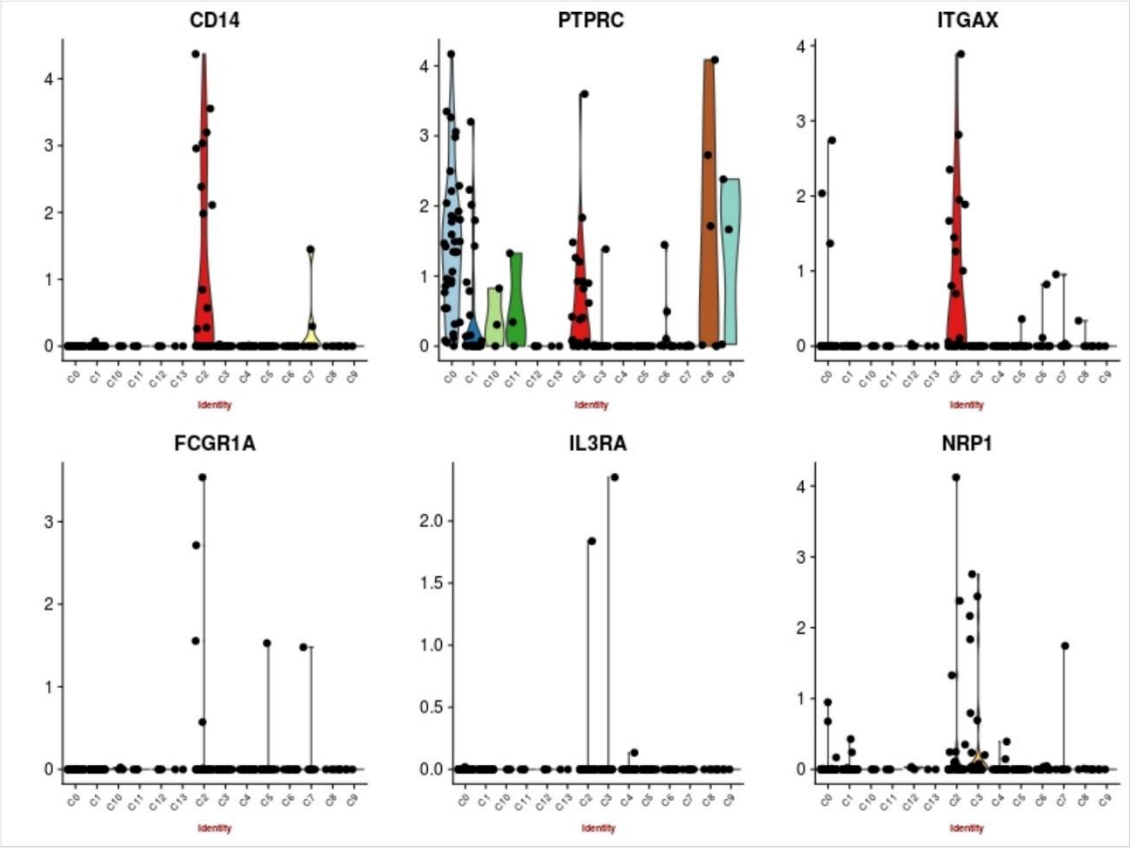

Supp. Figure 13

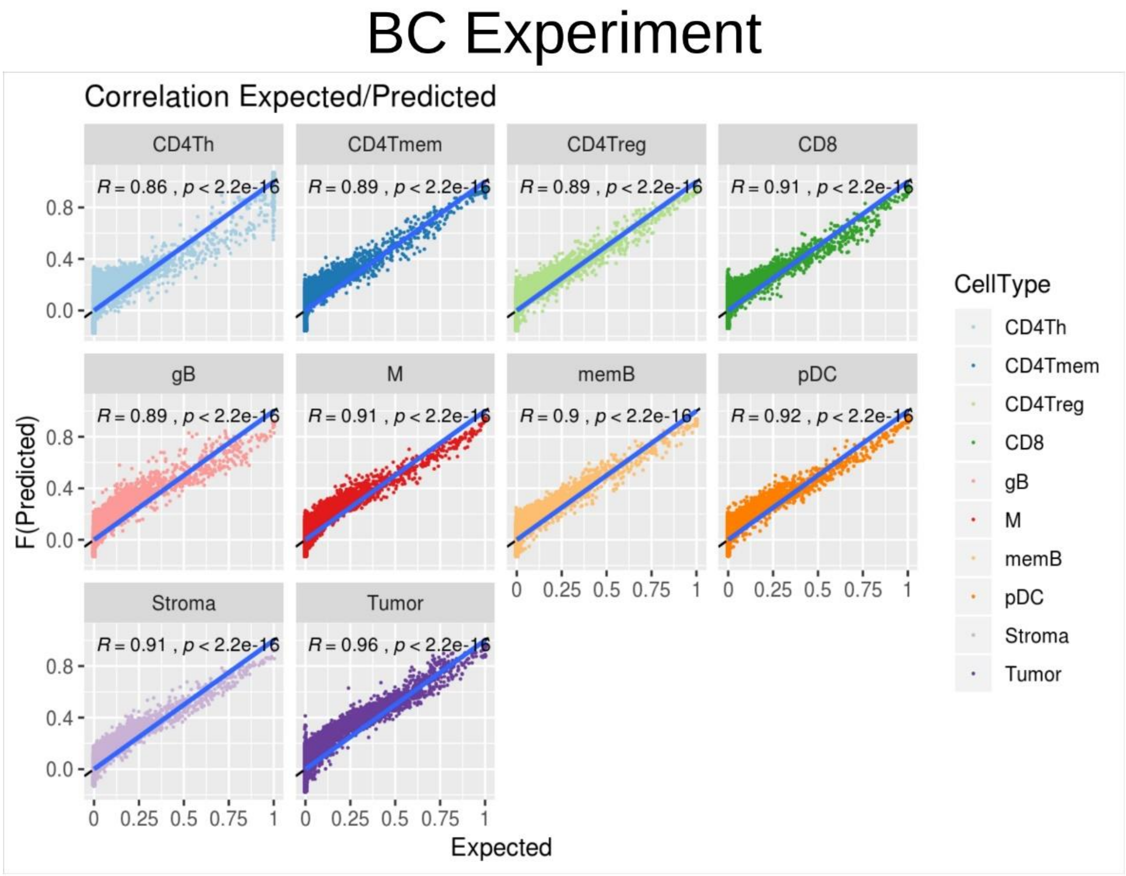

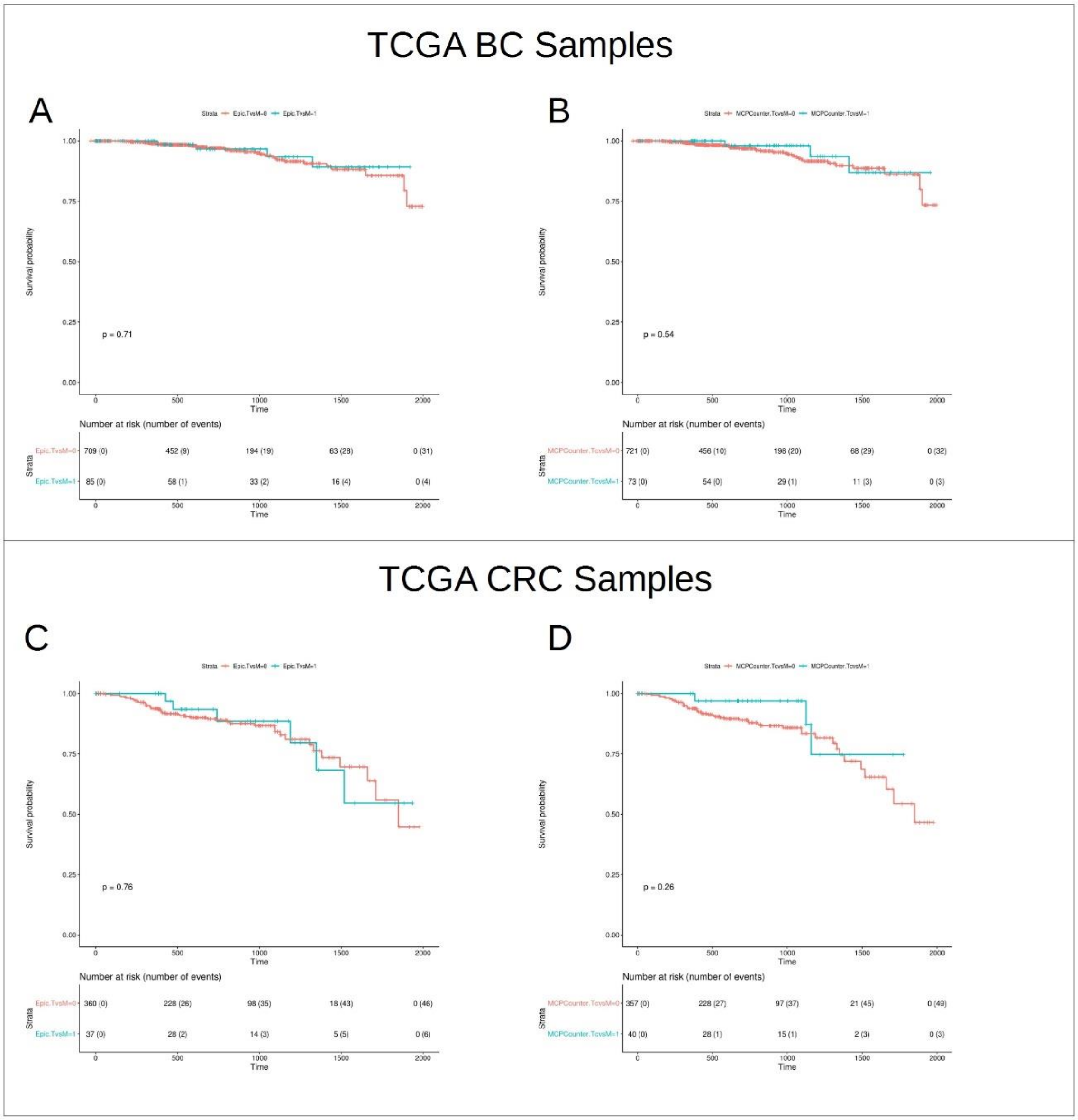

TCGA BC Samples
A
B
p = 0.71
p = 0.54
Survival probability
Time
Number at risk (number of events)
Strata
709 (0)
452 (9)
194 (19)
63 (28)
0 (31)
85 (0)
58 (1)
33 (2)
16 (4)
0 (4)
721 (0)
456 (10)
198 (20)
68 (29)
0 (32)
73 (0)
54 (0)
29 (1)
11 (3)
0 (3)
TCGA CRC Samples
C
D
p = 0.76
p = 0.26
360 (0)
228 (26)
98 (35)
18 (43)
0 (46)
37 (0)
28 (2)
14 (3)
5 (5)
0 (6)
357 (0)
228 (27)
97 (37)
21 (45)
0 (49)
40 (0)
28 (1)
15 (1)
2 (3)
0 (3)

Supp. Figure 1: tSNE representation of simulated and real cells from BC **(A)** and CRC **(B)** experiments. Colors indicate cell type. Solid dots label real cell profiles and open dots simulated profiles.

Supp. Figure 2: Bulk sample generation. **(A)** Box plots with the frequency distribution of each cell type produced from the different algorithms. Right column represents the frequency distribution of the vectors produced in the sets of the same row ordered from the highest to the lowest frequent in each vector. **(B)** Scheme of the bulk sample generation. For each frequency vector a 100 cells from the training or test set are sampled according to the frequencies and added to produce the bulk profile.

Supp. Figure 3: DigitalDLSorter training. **(A)** Scheme of the DNN model trained. **(B)** Progression of accuracy (acc), loss function (loss), Maximum absolut error (mae) and Maximum Absolut Proportional Error (log2(map)) during the 50 rounds of training process.

Supp. Figure 4: Grid Search Parameters. **(A)** Boxplot of normalized validation scores across generated models grouped by loss function. **(B)** Boxplot of Kullback-Liebler Divergence valudation metric grouped by number of neurons in the layers**. (C and D)** Boxplot of loss/val_loss metric grouped by dropout rate **(C)** or number of epochs **(D). (E)** loss versus val_loss meytrics coloured by number of epochs.

Supp. Figure 5. Hierarchical clustering heatmap representing the xCell scores (rows) across cells (columns) of the colorectal cancer experiment. Top annotations represents the cluster origin and the final cell type assignment.

Supp. Figure 6. Hierarchical clustering heatmap representing the xCell scores (rows) across cells (columns) of the breast cancer experiment. Top annotations represents the cluster origin and the final cell type assignment.

Supp. Figure 7. T Cells markers expression across clusters of the breast cancer experiment.

Supp Figure 8. B Cells markers expression across clusters of the breast cancer experiment.

Supp. Figure 9. Monocyte Cells markers expression across clusters of the breast cancer experiment

Supp. Figure 10. T Cells markers expression across clusters of the colorectal cancer experiment.

Supp. Figure 11. B Cells markers expression across clusters of the colorectal cancer experiment

Supp. Figure 12. Monocyte Cells markers expression across clusters of the colorectal cancer experiment.

Supp. Figure 13: Regression lines of the scaled square root of the predicted vs real proportions grouped by cell type.

Supp. Figure 14: Kaplan-Meier overall survival curves from breast (A and B) and colorectal (C and D) cancer patients using estimations from EPIC (A and C) and MCPCounter (B and D). In blue, samples within the highest 90th quantile of the ratio between T cells (CD8_TCells for EPIC, T_Cytotoxyc_Cells for

MPCCounter) over Monocytes/Macrophages (Macrophages for EPIC, Monocytic Lineage for MPCCounter). In red, individuals with low Tcells/Mono ratio.
